# ZnCo_(2−*x*)_Ce_*x*_O_4_, (*x*=0–1.0 wt%): optimized structural, morphological, dielectric, and magnetic properties for humidity sensing applications

**DOI:** 10.1039/d5na00355e

**Published:** 2025-07-02

**Authors:** Abdelfattah Darwish, Mohamed I. Farouk, Mohamed Morsy, Amir Elzwawy

**Affiliations:** a Microwave Physics and Dielectrics Department, Physics Research Institute, National Research Centre (NRC) 33 El-Bohouth St. Dokki Giza 12622 Egypt elzwawy1@gmail.com aa.elzwawy@nrc.sci.eg; b Civil Engineering Department, College of Engineering, Imam Mohammad Ibn Saud Islamic University (IMSIU) Riyadh 11432 Saudi Arabia; c Housing & Building National Research Centre (HBRC), Building Physics and Environment Institute Dokki Giza 12311 Egypt; d Ceramics Department, Advanced Materials Technology and Mineral Resources Research Institute, National Research Centre (NRC) 33 El Bohouth St. Dokki Giza 12622 Egypt

## Abstract

In this article ZnCo_(2−*x*)_Ce_*x*_O_4_ doped Ce was prepared by the sol–gel process where *x* = 0, 0.2, 0.5, and 1 wt%. The samples were investigated by XRD, SEM, FTIR, VSM, and dielectric studies. XRD shows the dominance of the cubic structure with an intense peak at 36°. SEM showed that the material changed from densely packed particles to flaky, porous structures. SEM results signify undoped samples having a pure ceramic microstructure with dense, well-packed particles. In contrast, doped samples reflect the residence particles and flaky features with more spaces between particles. FTIR showed two main peaks at 557 cm^−1^ and 660 cm^−1^ from Co–O and Zn–O stretching. When Ce was added, these peaks shifted and got weaker, showing that the structure became less ordered. The dielectric properties showed very high charge storage at low frequencies due to charges building up at boundaries. Ce doping made the charge storage lower and less sensitive to temperature. The combined para- and slight ferromagnetic nature is initiated with the highest cerium content (SCe1.0). The conductivity showed a flat region at low frequencies, and then increased following a power law. Ce doping made conductivity much lower. Sample SCe0.5 exhibited a distinctive conductivity feature because it had a good balance of trapped and free charges. Medium Ce levels (SCe0.2 and SCe0.5) led to balanced properties good for applications like supercapacitors and sensors. The application of the composite as a humidity sensor demonstrated high repeatability throughout a range of humidity levels (11–84% RH). The response and recovery times are 800 and 20 s, respectively.

## Abstract 2

In this article ZnCo_(2−*x*)_Ce_*x*_O_4_ doped Ce was prepared by the sol–gel process where *x* = 0, 0.2, 0.5, and 1 wt%.

## Graphical abstract

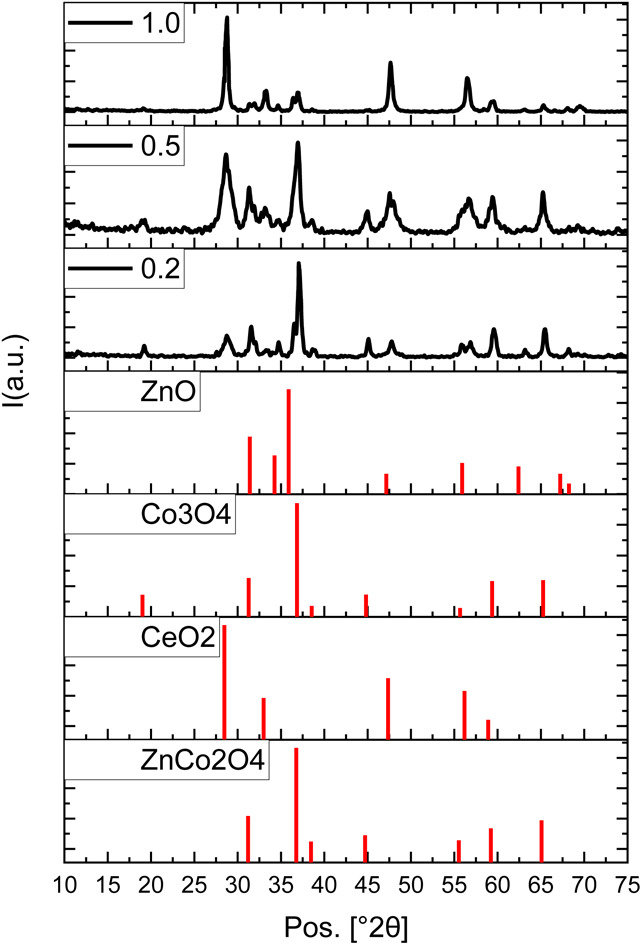

## 1. Introduction

Over the past few years, the issues related to controlling the amount of relative humidity have become vital as it is related to human beings and modern industries^1,2^ The quantification of relative humidity requires highly sensitive and fast response humidity sensors.^3,4^ These key parameters can be achieved using nanomaterials with special functional properties.^4^ Many materials were investigated to be used in humidity sensing applications. Metal oxides, ceramics, perovskite, polymers, and others have been explored for humidity sensing applications,^5–7^ focusing on the advantages of metal oxides and ceramics that include low cost, abundance, ease of fabrication, and morphology and structure tuning, in a wide range of applications. The utilization of metal oxides and ceramics offers a straightforward strategy for getting highly efficient humidity sensors. Regarding the humidity sensing applications, the key issues related to the response time and sensitivity need to be addressed. Regardless of the outstanding functional properties of metal oxides and ceramics for humidity sensing applications, the response time and sensitivity present an obstacle. To overcome these limitations, many strategies were developed and investigated.^8,9^ Binary transition metal oxides like ZnCo_2_O_4_, NiCo_2_O_4_, CuCo_2_O_4,_ and ZnFe_2_O_4_ have received much attention over the past decade. Despite being a p-type semiconductor, zinc cobaltite (ZnCo_2_O_4_) exhibits a spinel-type structure with a band gap energy of 2.6 eV, and has been used in many applications.^10,11^ Zinc cobaltite (ZnCo_2_O_4_) is inherently employed in supercapacitors and energy storage devices,^12,13^ photocatalysis and solar cells,^14,15^ sensing areas,^9,16–20^ and antibacterial and biomedical applications.^21^

Zinc cobaltite (ZnCo_2_O_4_), with its steady spinel structure, provides a variety of appreciated properties that make it highly appropriate for industrial applications. Its combined-valence nature and tunable cation distribution allow desirable magnetic and electrical characteristics, making it beneficial in magnetic sensors, spintronic devices, and electronic components. The material also exhibits admirable electrocatalytic activity and redox behavior, which are advantageous for applications in energy storage systems such as lithium-ion batteries and supercapacitors. Moreover, its semiconducting properties and elevated surface area permit effective gas sensing and photocatalytic activity, making it valuable in environmental monitoring and pollutant degradation. ZnCo_2_O_4_'s increased thermal and chemical stability further supports its usage in harsh environments, including in protective coatings and catalyst supports. These multifunctional properties, conjugated with the potential for scalable synthesis, highlight zinc cobaltite as a promising material for a wide range of technological and industrial applications.^14,15,22,23^ Many synthesis routes such as co-precipitation, sol–gel, combustion, microemulsion, thermal decomposition, and hydrothermal were utilized to prepare ZnCo_2_O_4_ (ref. 24, 25 and 26).

Preeti Lata Mahapatra *et al.*,^27^ prepared a cobalt chromite-based humidity sensor using the sol–gel method. The sensor exhibits a fast response and recovery time at 5–95% RH. Ebtesam E. Ateia *et al.*,^28^ synthesized a LaCoO_3_ mesopore sensor by a modified citrate technique and inspected the humidity sensing performance within the range of 11–97% RH, and the frequency range of 100 Hz–100 kHz. The attained results confirm that the optimum measuring frequency is 1 kHz. A humidity sensor based on bifunctional NiCo_2_O_4_/g-C_3_N_4_ was presented by Likun Gong *et al.*,^29^ showing a noteable sensitivity of 1471 kΩ/% RH@11% RH. Cobalt ferrite nanoparticles (CoFe_2_O_4_) with precise morphology were attained *via* a solution route.^30^ The highest humidity sensitivity value of ∼590, along with a response/recovery value of 25/2.6 s at room temperature, was obtained. ZnCr_2_O_4_–ZnO composites were studied for humidity sensing. The composites were subjected to DC resistance measurements as a function of relative humidity within the range of 5–98% RH. The material exhibited high sensitivity, good linearity, and reversible characteristics.^31^ ZnAl_2_O_4_/Al was also applied for humidity sensing. All fabrication processes were conducted at 400 °C, where the ZnAl_2_O_4_/Al device exhibits decent sensitivity and considerable repeatability as a humidity sensor.^32^ The humidity sensing is evaluated using Ni_*x*_ Cu_0.8−*x*_ Zn_0.2_Fe_2_O_4_, 0.0 ≤ *x* ≤ 0.8 (*x* = 0, 0.2, 0.4, 0.6, 0.8) in the range of 5–98% RH. *x* = 0.4 possessed the highest humidity sensing factor of 3051.9 ± 500.^33^

To the best of our knowledge, the research work devoted to utilizing ZnCo_2_O_4_ as a humidity sensor is limited. Starting from the research gap, in this research, the ZnCo_2_O_4_ nanostructures doped with different ratios of Ce were synthesized by the sol–gel method. The nanomaterials obtained were characterized through XRD, SEM, FTIR, VSM, and dielectric studies. The humidity sensing behavior of the synthesized materials was investigated in a wide range of relative humidities (11–97%) using a saturated salt solution. The sensitivity, hysteresis, response, and recovery times were also investigated. Common dopants like Ni, Cu, and Mn have been used but do not fully solve the instability problem. Ce is a rare-earth element with a large ion size [(Ce^4+^ = 0.97 Å) compared to Co^3+^ (0.61 Å)] which changes the structure and electrical properties. Furthermore, it provides oxygen vacancies and metal–oxygen bonds and controls the charge movement. The doping is expected to lead to an enhancement in both response and recovery times for potential industrial applications beneficial for the future era. The potential route is beneficial for the nanotechnological applications applying humidity sensing devices in a wide spectrum.

## 2. Experimental section

### 2.1 Raw materials used for synthesis

Cobalt nitrate hexahydrate (Co(NO_3_)_2_·6H_2_O) was obtained from Sigma Aldrich along with zinc acetate ((CH_3_CO_2_)_2_Zn). Cerium was introduced in the intended molar ratio from the initial precursor cerium nitrate hexahydrate (Ce(NO_3_)_2_·6H_2_O) purchased from Fluka. Other chemicals encompassing citric acid and acetic acid are provided by Sigma Aldrich. The functioned chemicals are employed as received without prior treatment.

### 2.2 The preparation approach of ZnCo_(2−*x*)_Ce_*x*_O_4_

**Scheme 1:**
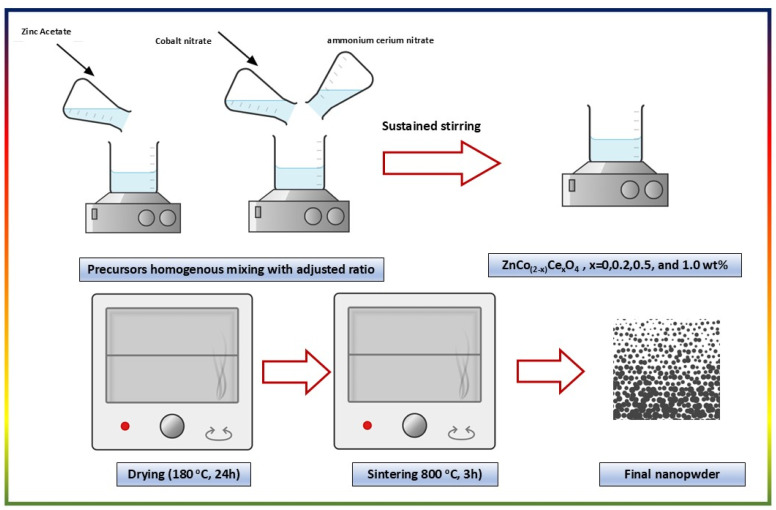
The synthesis approach for ZnCo_(2−*x*)_Ce_*x*_O_4_, (*x* = 0–1.0 wt%).

The experimental approach follows a feasible sol–gel procedure. Initially, separate precursor solutions were prepared for the Zn, Co, O, and Ce elements. The final ratio and molecular weight of the elements were considered. Ce was added in a %mol, replacing the Co in the network structure. The spinel structure of the Zinc cobaltite znCo_(2−*x*)_Ce_*x*_O_4_ was affirmed, where *x* = 0, 0.2, 0.5, and 1 wt%. The samples are named SCe0, SCe 0.2, SCe0.5, and Sce 1.0 based on the Ce content. A few points were highlighted to verify a better dissolution of the precursors. Zinc acetate was dissolved in a mixture of distilled water and glacial acetic acid (4 : 1 respectively), and calculated amounts of cobalt nitrate and ammonium cerium nitrate were dissolved in distilled water and were then combined under stirring. After sustained stirring for one hour, citric acid was added to the zinc acetate solution during stirring in a molar ratio of 3 : 1 to zinc, cobalt (and cerium) ions, and then the cobalt and cerium precursor solution mixture was added too. The stirring is continued for an additional 4 hours. The formed gel was fired at 180 °C, for 24 h, and then sintered at 800 °C for three hours. Finally, the prepared nanopowders are ready for further investigation. The scheme for the preparation is introduced (Scheme 1).

### 2.3 Measurement devices

The prepared nanopowders were characterized by using a set of devices. The diffraction peaks were obtained by XRD using a Malvern Panalytical Empyrean 3 diffractometer with a CuKα radiation source (*λ* = 1.5406 Å) in the scanning range of 10–75°, maintaining a 2° per minute scanning rate. The morphological features and elemental mapping analyses were acquired using a SEM instrument (Quattro S, Thermo Scientific model), which functioned at 20 kV. The functional group interaction and fingerprint distinguishable bands were attained using FTIR (Vertex 70, Bruker) within the spectral range of 4000–400 cm^−1^ verifying a 4 cm^−1^ spectral resolution. A Vibrating-Sample-Magnetometer (VSM: 7410 Series-Lake Shore Cryotronics, USA) was used to explore the magnetic behavior. The dielectric specifications were realized by employing broadband dielectric spectroscopy throughout a wide range of frequencies (10^−1^ to 10^7^ Hz) and temperatures between 30 and 150 °C with a 20 °C step. The technique employs an increased resolution ALPHA analyzer with an active sample holder head (Model: Novocontrol, Montabaur, Germany), conjugated with an active sample head. A Quatro temperature controller system was utilized for the stabilization of temperatures lower than 0.2 K, and pure nitrogen was applied as a heating agent.

### 2.4 Humidity sensor fabrication and evaluation

Humidity sensors were fabricated using a simple drop-casting method. In brief, an appropriate amount of sensitive materials is mixed with a minimum amount of 2% PVA solution to form a paste. PVA was used as a binder. The formed slurry was deposited over a fluorinated tin oxide (FTO) coated glass substrate *via* the doctor-blading method, resulting in a uniform film with an approximate thickness of 450 μm and dried at 150 °C for three hours. The fabricated sensor was aged at low and high humidity levels for 24 hours at each level. The impedance values of the fabricated sensor were measured at different frequencies using an HIOKI 3532 LCR meter. The optimum testing frequency was determined based on the maximum variation in impedance when the sensor was subjected to different values of relative humidity. The desired level of relative humidity was generated using a saturated salt solution. The general guidelines of the ASTM e104 standard have been followed for this purpose. The sensor was allowed to settle at each humidity level for at least 15 minutes, and then the corresponding impedance value was recorded.

## 3. Results and discussion

### 3.1 XRD investigation

**Fig. 1:**
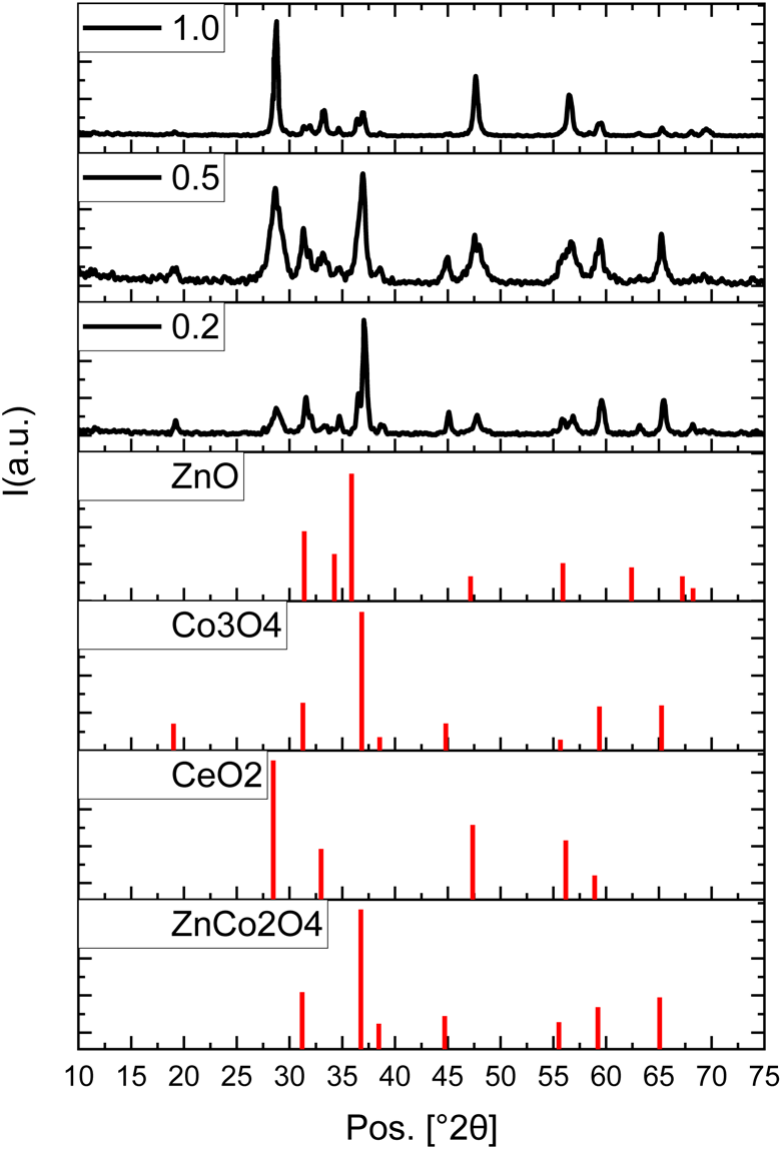
The diffraction pattern of the prepared ZnCo_(2−*x*)_Ce_*x*_O_4_, *x* = 0.2, 0.5, 1.0, along with the ICSD diffraction cards.

The diffraction pattern of the ZnCo_(2−*x*)_Ce_*x*_O_4_ nanopowders is demonstrated in Fig. 1. The samples are named SCe0.2, SCe0.5, and SCe1.0, based on the Ce content. The dominant phase and diffraction pattern is ascribed to the cubic structure zinc cobalt oxide ZnCo_2_O_4_ following the ICDD card no. 01-088-3763 matching the former results.^34^ The crystallographic lattice parameters are *a* = *b* = *c* = 8.1 Å, while *α* = *β* = *γ* = 90°. The main peaks for ZnCo_2_O_4_ are located at 31°, 36°, and 65°, which are attained from the diffraction planes (220), (311), and (440), respectively, as illustrated in Fig. 1. The ZnO hexagonal phase is present as well with overlapping peaks matching the ICSD card no. 01-086-8199 and former research^35,36^ Besides, the Co_3_O_4_ cubic structure with the ICSD card no. 01-086-8289 also reinforces the preceding peaks, especially the most intense one at around 36°.^37^ The inclusion of the propagated ratio of cerium into the network of zinc cobaltite is revealed. Initially, upon the reduced incorporated portion of the cerium, a small peak near 28°, which somehow displays lower intensity in the reduced Ce ratio (<1%), which is conversely increased afterward. This is acceptable as this is the main peak of cerium oxide. The cerium oxide main characteristic peaks are visualized as per the ICSD card no. 01-085-9125. Other peaks of cerium oxide propagate with the increase of Ce doping into zinc–cobaltite as the peak near 47°, 56°.

### 3.2 SEM

**Fig. 2:**
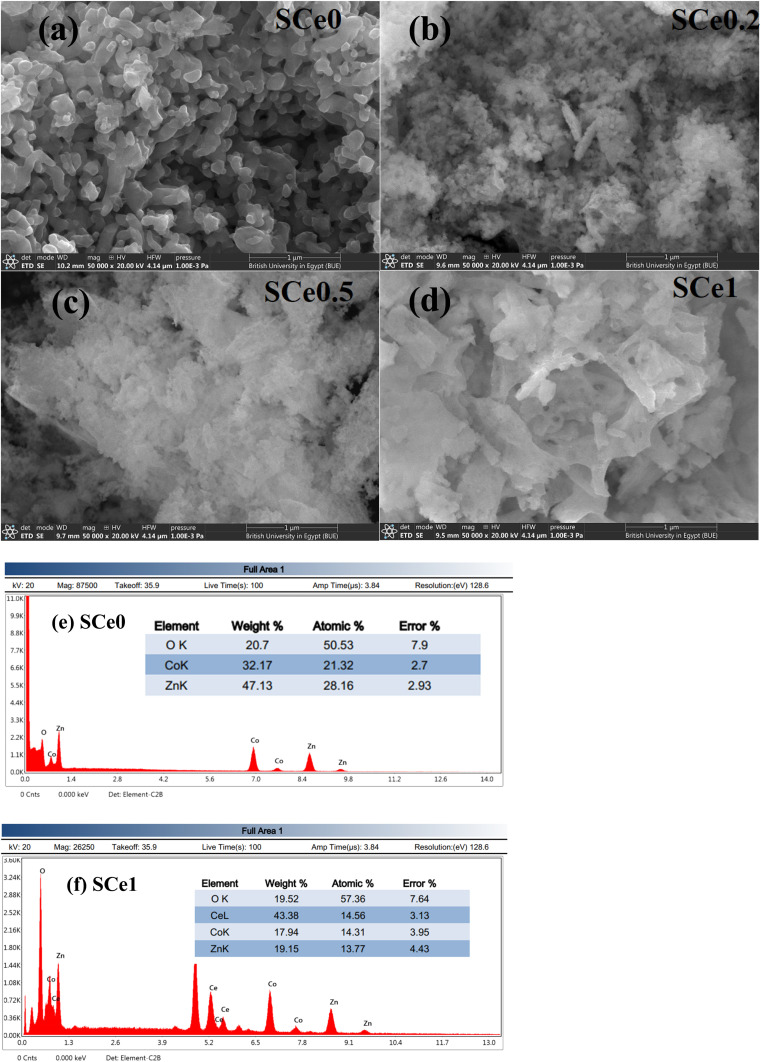
SEM images of all samples ZnCo_(2−*x*)_Ce_*x*_O_4_, *x* = 0, 0.2, 0.5, and 1.0 denoted as SCe0, SCe0.2, SCe0.5, and SCe1.0 respectively (a–d) and (e) and (f) EDX for SCe0 and SCe1.0, respectively.

The sample morphology is depicted in Fig. 2. Sample SCe0 has a pure ceramic microstructure with dense, well-packed particles. The particles have clear boundaries between them and smooth surfaces. The particles are uniform in size and show limited porosity. They are well-connected to each other. Sample SCe0.2 shows the beginning of structure changes. It has a mix of particles and flaky features, with more spaces forming between particles. The surface gets rougher, and the structure becomes less uniform than that of SCe0. Some particles break into smaller pieces, and pores start to form, though they retain some original particle nature. Sample SCe0.5 shows a clear transformation to a flaky structure. It is much more porous than SCe0.2, and irregular shapes become common. The flakes stack on each other with more open spaces between them. The surface is rougher, and the structure is less dense, showing a clear break from the original particle nature. Sample SCe1 shows a complete transformation into a flaky, sheet-like structure. It has many small flakes and is very porous. The flakes are oriented randomly and create a high surface area. There are clear void spaces and no trace of the original particle structure, making it look almost sponge-like. As Ce content increases, there is a transformation from particles into flakes. The structure gets more porous with more irregular shapes and less dense packing. Surface area increases with more void spaces. There is less particle connectivity, and the structure becomes more complex. These structural changes affect properties. More pores make it harder for charges to move. The flaky structure creates more surface area. Less connectivity leads to lower conductivity. More boundaries trap more charge. The complex structure creates multiple relaxation times. More voids lower overall permittivity. Surface changes affect charge dynamics. The structure explains changes in electrical properties. The features make these materials good for applications. The doped samples have high surface area and good porosity. The flakes have many active sites. These qualities make the materials good for gas sensing, catalyst supports, and supercapacitors. They allow enhanced surface reactions and better interaction between the material and the environment.

### 3.3 FTIR

**Fig. 3:**
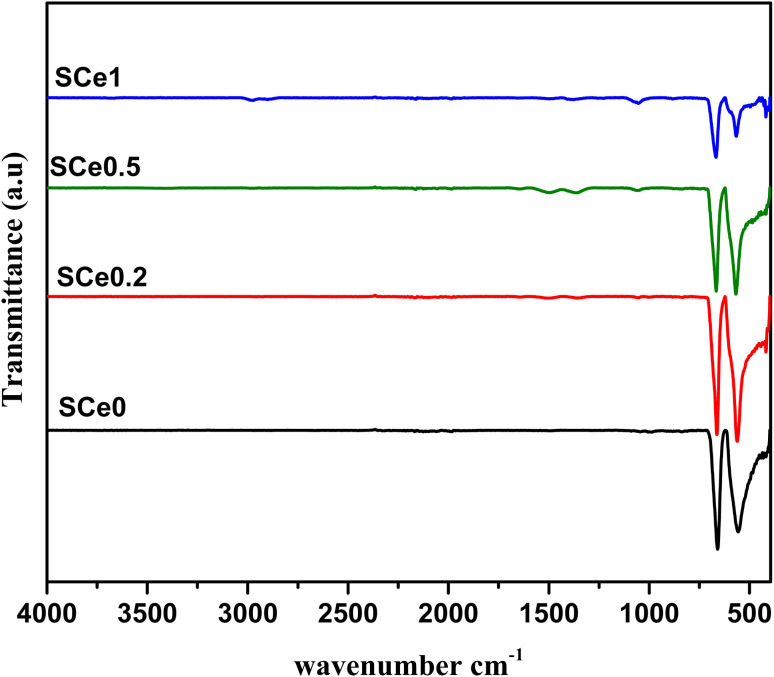
FTIR spectra of SCe0.2, SCe0.5, and SCe1 composites in the 4000–400 cm^−1^ wavenumber range.

The spectral lines stay mostly flat from 4000 to 1000 cm^−1^ (Fig. 3). Two main sharp absorption bands are seen at 557 and 660 cm^−1^. The 557 cm^−1^ band comes from Co–O stretching in tetrahedral sites. The 660 cm^−1^ band comes from Zn–O stretching in octahedral sites. The bands shift, and their intensity decreases progressively with Ce addition. The decrease in intensity means the structure is becoming less ordered, and the shifts mean the lattice is changing. Inspection of the SEM images shows that the pure sample SCe0 has dense and well-packed particles with clear boundaries and smooth surfaces, while the structure of the Ce-containing samples turns progressively into a flaky form with much more porosity. These structural changes affect the metal–oxygen bonds. As the structure becomes more porous and disordered, the bond lengths and angles can change. This may cause the absorption bands to decrease in intensity or shift slightly. The formation of oxygen vacancies when Ce replaces smaller ions can also change the bond environment. Increased disorder and lattice strain weaken the metal–oxygen bonds. This explains why the FTIR peaks change when more Ce is added.

### 3.4 VSM

**Fig. 4:**
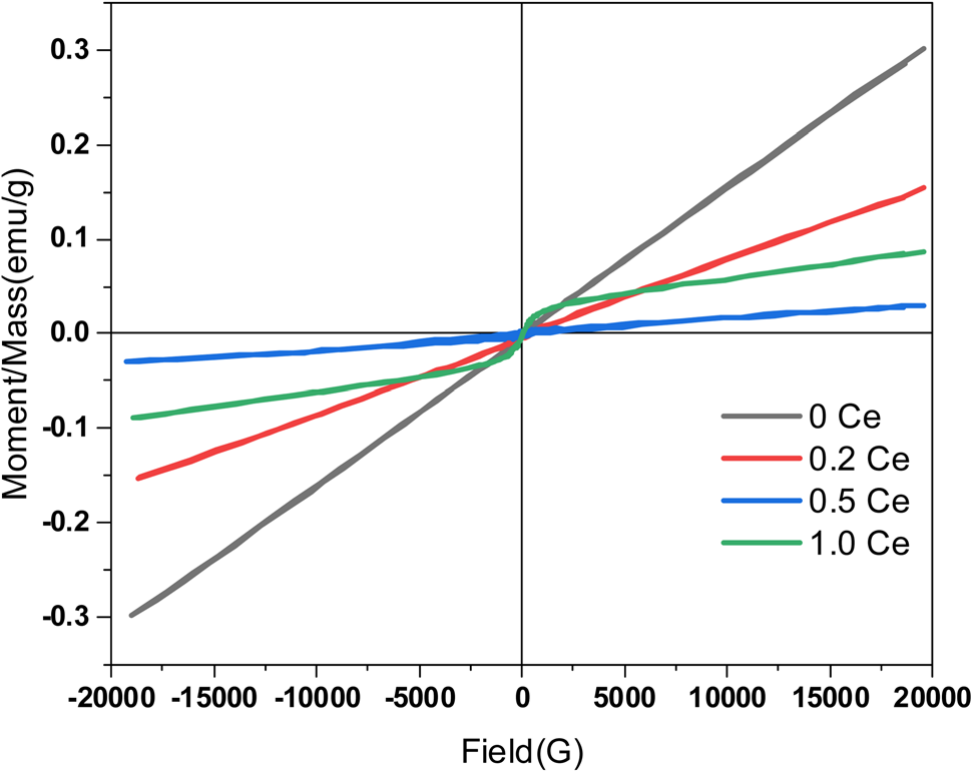
The measured hysteresis loop of the prepared ZnCo_(2−*x*)_Ce_*x*_O_4_ with an altered cerium doping ratio. The measurements were acquired within a sweeping alternating magnetic field of ±20 kG under ambient conditions.

**Fig. 5:**
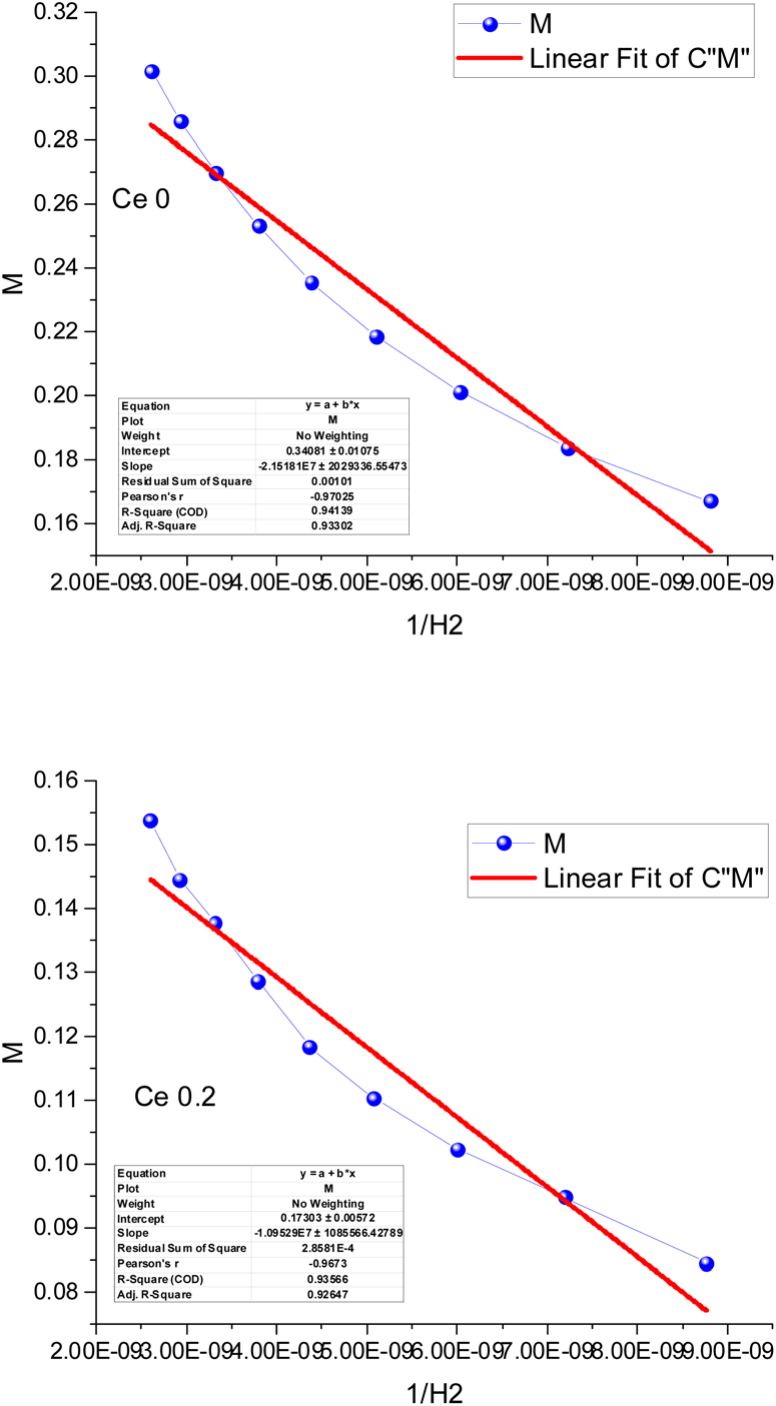
The determination of the theoretical magnetization following the law of approach for varied Ce contents in the ZnCo_(2−*x*)_Ce_*x*_O_4_ nanostructure (Ce0, and Ce0.2).

Throughout the past few decades, a vibrating sample magnetometer has emerged as a device for evaluating the magnetic merits of the provided nanomagnetic materials.^38–41^ Commonly, prominent classes of magnetic behavior are referred to as ferromagnetic, antiferromagnetic, paramagnetic, and superparamagnetic. Each of these behaviors exhibits a distinct hysteresis loop shape.^42^ A hard ferromagnetic material has large coercivity and saturation, while soft ferromagnetic material exhibits reduced coercivity. Superparamagnetic materials have diminished coercivity and remnant magnetization accompanied by increased saturation magnetization.^43,44^Fig. 4 presents the vibrating sample magnetometer (VSM) results for ZnCoFe_2_O_4_ obtained at room temperature through a spacious operating field −20 kG to 20 kG. The first glance at the hysteresis loop of the samples reveals a paramagnetic tendency, while a straight line with a positive slope is remarked without the offered saturation confirmation even at upsurged magnetization values. This tendency is more characteristic in the undoped sample (0 Ce) and the tiny doped sample (0.2 Ce). The initial permeability is increased in the undoped sample, reflected by the elevated slope magnitude. Upon the inclusion of higher cerium content in the polymeric matrix of the composite, a slight ferromagnetic behavior starts to arise, accompanied by the upsurge of the coercivity magnitude. The obtained magnetic parameters from the hysteresis loop are usually the saturation magnetization (*M*_S_), the coercivity (*H*_C_), the remanent magnetization (*M*_r_), the squareness ratio (SQ), and others. Theoretically, saturation magnetization can be obtained when all the magnetic domains are aligned following the externally applied magnetic field, and thus, the upper section of the hysteresis loop approaches a plateau. The optimum value of this plateau represents the saturation magnetization (Fig. 5).

In the case of the paramagnetic behavior, no experimental saturation magnetization can be acquired; instead, a theoretical saturation value can be extrapolated following the law of approach (LOA) to saturation.^38,45^ Through the relation between 1/H^2^ and the corresponding *M* value, we can obtain the theoretically estimated saturation magnetization magnitude.^46^ Inherently, the plot is drawn for the high field range (>10 kG). Fig. 5 presents the determination of the saturation magnetization of the samples. The theoretically acquired values of *M*_S_ are 0.34 emu g^−1^ and 0.17 for Ce0 and Ce 0.2, respectively, as displayed in Fig. 5.

### 3.5 Dielectric study

#### 3.5.1 The dielectric permittivity

**Fig. 6:**
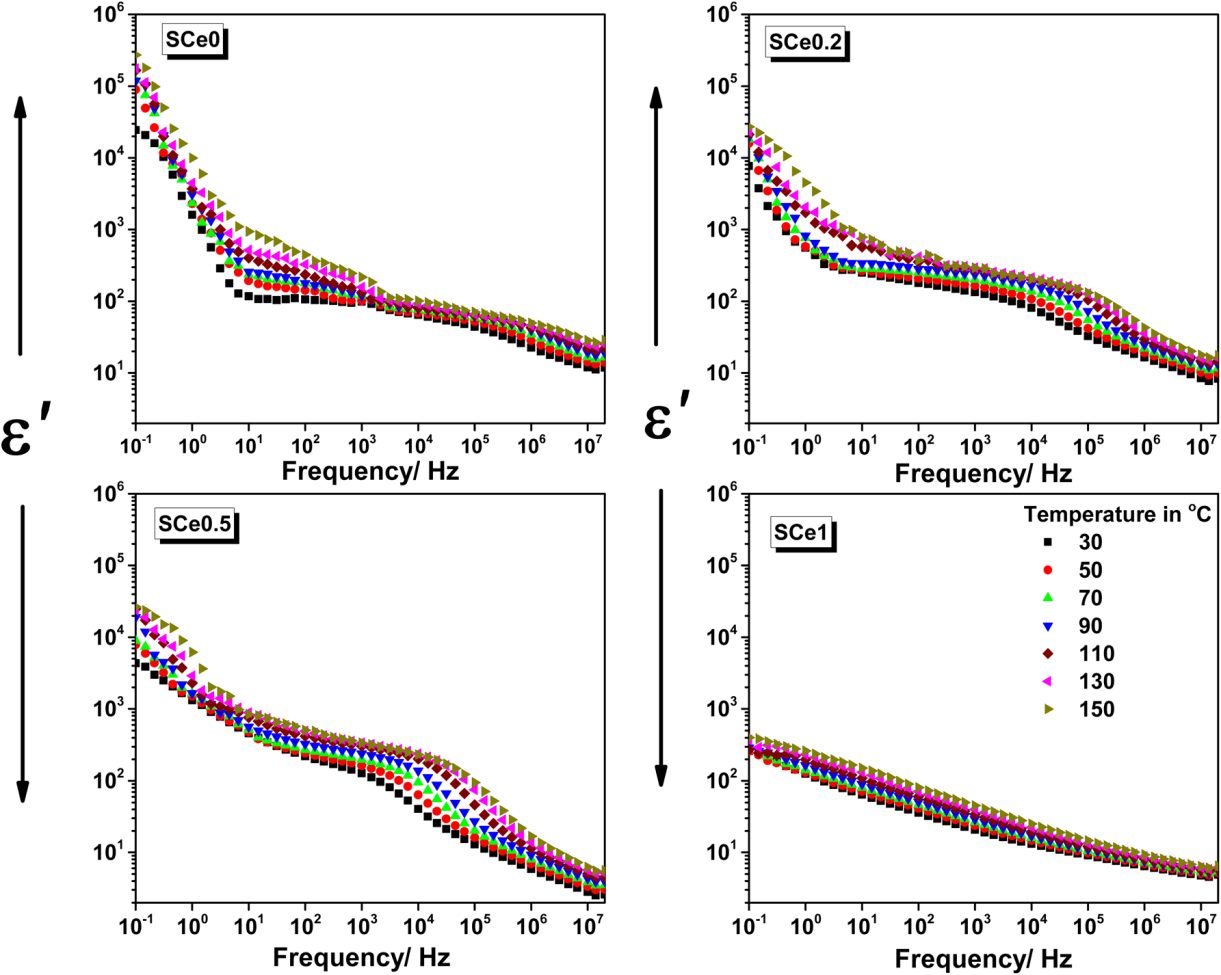
The dielectric permittivity *vs.* frequency for all samples at different temperatures as indicated, from 30 °C to 150 °C, in steps of 20 °C.

The samples show different permittivity behaviors in their dielectric response (Fig. 6). At low frequencies from 0.1 to 10 Hz, the permittivity values are the highest, where the neat sample SCe0 at a temperature of 30 °C and at a frequency of 0.1 Hz starts with the highest permittivity around 2.5 × 10^4^, and then the permittivity values get lower as more dopant is added, and sample SCe0.2 shows about 7.7 × 10^3^, SCe0.5 about 4.4 × 10^3^, and SCe1 shows about 260. The frequency response shows an initial steep drop, followed by a flatter region that looks like a plateau from 10 Hz to 10 kHz. This plateau is very clear in SCe0 and SCe0.2 but starts to disappear with more doping. In SCe0, this plateau shows a slight negative slope, becoming more visible at higher temperatures. SCe0.2 maintains this plateau but with a steeper slope. After 10 kHz, there is a second drop in the permittivity, which is very clear in SCe0.2 and SCe0.5 but not visible in SCe1. Temperature affects the samples differently. SCe0 shows the strongest temperature effect, especially between 1 and 100 Hz, showing about a one order of magnitude difference between 30 °C and 150 °C and reaches 2.7 × 10^5^ at a frequency of 0.1 Hz. The second dispersion is more sensitive to temperature than the first drop in samples SCe0.2 and SCe0.5. The sample SCe1 barely changes with temperature at all; it shows minimal temperature sensitivity, with curves almost overlapping across all frequencies.

When more dopant is added, the overall permittivity values get lower, the curves become smoother, and temperature has less effect. The total permittivity drops from the lowest to the highest frequency is about four orders for SCe0, three orders for SCe0.2 and SCe0.5, and less than two orders for SCe1. The overall behavior changes from a clear two-step decrease with a plateau (SCe0) to an almost linear decrease (SCe1), showing how doping smoothens the dielectric response. SCe0 and SCe0.2 retain their two-step decrease and clear plateau region. SCe0.5 shows a behavior in between, where the regions start to blend together. SCe1 is very different – it shows an almost straight line decrease with frequency, stays below 10^3^ in permittivity, and doesn't change much with temperature. At very high frequencies, all samples end up having low permittivity values.

The dielectric permittivity behavior changes with frequency, temperature, and Ce content which can be explained as follows.^47^ At low frequencies (0.1–10 Hz), charges pile up at grain boundaries, causing high permittivity (Maxwell–Wagner effect).^48^ This occurs because charges have time to move and pile up at interfaces. The neat sample SCe0 shows high permittivity (2.5 × 10^4^ at 30 °C) because it has many grain boundaries. Adding Ce drops the permittivity (7700 for SCe0.2, 4400 for SCe0.5, and 260 for SCe1) by reducing boundaries and defects. Heat makes charges move more easily, so temperature strongly affects this region. For example, SCe0 at 0.1 Hz increases its permittivity from 2.5 × 10^4^ at 30 °C to 2.7 × 10^5^ at 150 °C because charge carriers gain more energy to move to interfaces.

In the frequency range of 10 Hz to 10 kHz, there is a plateau where permittivity changes slowly. Here, three things occur together: charges hop between sites, dipoles rotate, and some charges still build up at interfaces. These processes overlap because the frequency is not too high or too low. The plateau slopes down because the material loses some energy moving charges. This energy loss occurs because dipoles and charge carriers dissipate some energy as heat when trying to reorient or hop between sites. The heat helps charges to hop, and the dipoles rotate, making the plateau more visible at high temperatures. The plateau becomes less clear with more Ce because Ce reduces places where charges can move and makes dipoles less flexible.

After 10 kHz, SCe0.2 and SCe0.5 show a second dispersion in permittivity. This occurs because dipoles can't turn fast enough to follow the electric field. The heat helps dipoles move more easily, so temperature strongly affects this region. SCe1 doesn't show this drop because Ce makes the structure very stiff and rigid, limiting dipole movement, which is apparent from SEM analysis and the structure mutation from particle nature into the flaky structure in the doped samples. In sample SCe1, Ce ions replace Co and disrupt the normal dipole behavior, making the remaining dipoles too constrained to contribute to relaxation. The high concentration of Ce also traps oxygen vacancies, preventing charge carriers from contributing to the second dispersion process. When Ce^4+^ (0.97 Å) replaces Co^3+^ (0.61 Å), it changes the structure because Ce^4+^ is bigger, as Ce^4+^ pulls oxygen atoms closer due to its higher charge, squeezing the local structure.^49–51^ The material makes oxygen vacancies to balance the extra charge of Ce^4+^. Ce makes the structure flaky with more pores, which cuts connections between grain boundaries. Ce–O bonds (1.8–2.2 Å) are longer than Co–O bonds (1.6–1.9 Å).^52^ These different bond lengths affect how charges move in the material. When charges try to move back and forth (dipole movement), the stretched and squeezed bonds make it harder.^52,53^ That is why samples with more Ce show lower permittivity and react less to temperature. At very high frequencies, only electron clouds around atoms can follow the field. Larger charges and dipoles are too slow to respond. This is why all samples show similar low permittivity values at high frequencies. The overall behavior shows how the material switches from interface effects at low frequencies to mixed effects in the middle, to just electron movement at high frequencies. Temperature affects each region differently based on how easily charges and dipoles can move in that frequency range.

#### 3.5.2 The AC conductivity

**Fig. 7:**
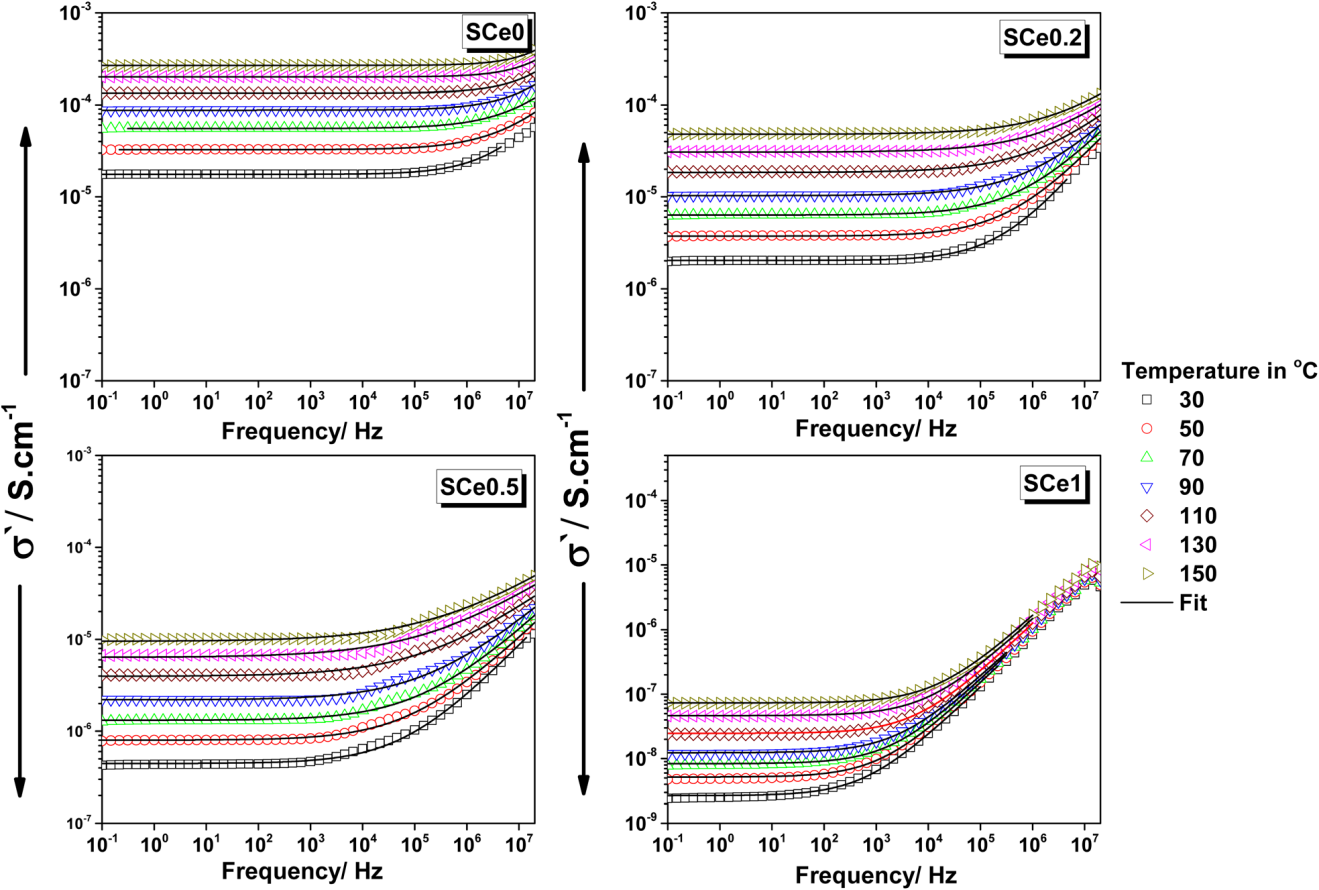
The AC conductivity (*σ*′) *vs.* frequency for all samples at different temperatures as indicated, from 30 °C to 150 °C, in steps of 20 °C.

The samples show different conductivity patterns as frequency and temperature change (Fig. 7). SCe0 conductivity stays flat from 0.1 Hz to 1 kHz, then starts increasing above 1 kHz. The increase gets steeper above 10 kHz, following a power-law shape. The conductivity of SCe0 at low frequencies stays around 10^−5^ S cm^−1^ and reaches 10^−3^ S cm^−1^ at high frequencies. Temperature changes are more robust in the flat region. The curves show clear steps up with temperature increment. The temperature effect gets smaller at high frequencies, where the curves come closer together. The lowest values are at 30 °C and the highest at 150 °C, showing about a 100 times increase.

Adding Ce promotes consistent lessening to the conductivity. Sample SCe0 has the highest values, and then SCe0.2 drops to about one-third that of SCe0. SCe0.5 shows another big drop, and SCe1 has the lowest values, about 10^4^ times lower than that of SCe0. The flat parts shrink to a lesser frequency span with more Ce content, and the higher frequency parts show more converging behavior. Temperature effects get smaller with more Ce content. The critical frequency at which conductivity starts following the power law rather than dc conductance behavior shifts to higher frequencies on increasing either Ce content or temperature. SCe0.5 shows something special – a bump or a peak in the middle frequencies. This bump moves from 10 to 100 kHz when the temperature goes up. The bump size changes with temperature and is the biggest at moderate temperatures. No other sample shows this bump feature. Each sample has its own conductivity range, where each range delivers about 100 times change across frequencies.

The conductivity trends are explained as follows. The steady-state and frequency-independent behavior of conductivity that appears as a plateau at low frequencies can be attributed to charges building up at grain boundaries. The charges accumulate and get trapped within these boundaries, blocking the current flow. When charges get trapped at grain boundaries, the permittivity increases. This means the material can store more electric energy. The trapped charges change both how current flows and the dielectric properties. They make the local electric field stronger. The grain boundaries help make the dielectric response stronger and promote interfacial polarization. The charge buildup affects permittivity, which shows up as higher permittivity values at lower frequencies, followed by an abrupt drop, as seen in the samples.

Heat makes charges move more easily in the material and helps trapped charges get out from grain boundaries. This causes the conductivity plateau to rise since charges acquire more mobility. By heating, the critical frequency shifts to higher values. Heat reduces charge trapping, so power-law behavior starts at higher frequencies. Heating reduces how much grain boundaries affect charges as the charges move faster with heat, which delays the change from DC to power-law behavior. The relaxation time becomes shorter, which makes the critical frequency shift to higher values. The power-law region is dominated by intrinsic material properties and relies on bulk charge dynamics, so it shows less sensitivity with heating and is affected less by grain boundaries.

The overall conductivity decreases with Ce doping, which can be attributed to its impact on the structure, lattice strains, and conduction pathways. As mentioned earlier in the permittivity discussion, the larger size of Ce^4+^ and the longer bond with oxygen not only distorts the structure and causes lattice strains but also disrupts charge pathways and makes the structure flaky with more pores, which cuts connections between grain boundaries. All of these pile up as negative impacts on conductivity. Furthermore, the oxygen vacancies created to compensate for charge difference are not helping in conduction, but they may trap charges and reduce the mobility instead of aiding conduction. Ce doping reduces the flat part of conductivity because it disrupts grain boundaries. The trapped charges at grain boundaries decrease due to structural changes and strain. With fewer trapped charges, less charge accumulates at the boundaries, which makes the flat region smaller. The critical frequency shifts to lower values because there is less trapping. When fewer charges are trapped, the power-law behavior starts earlier. Ce doping weakens the effects of grain boundaries, which allows the transition to occur at lower frequencies. Overall, the flat part becomes smaller, and the critical frequency moves to lower values because of reduced trapping and weaker grain boundary effects.

The bump or the peak noted in SCe0.5 is due to special charge behavior at moderate Ce doping. Moderate Ce doping creates a balance between trapped charges and free carriers or may be due to a balance of charge hopping and dipole rotation. At moderate frequencies, trapped charges start to move, causing extra conduction. The bump moves from 10 to 100 kHz with temperature because heat speeds up the movement of charges and dipoles. The size of the bump changes with temperature. At low temperatures, fewer charges move, and at high temperatures, all charges are already moving, so the bump disappears. No other sample shows this because SCe0.5 has a unique balance of Ce^4+^ effects. Other samples do not have this specific doping level to create the bump. The bump is caused by charge release at grain boundaries at moderate frequencies. Temperature affects the position and size of the bump because it changes how charges move. SCe0.5 is unique because moderate Ce doping creates the right conditions for this effect.

Overall, transport mechanisms show three distinct frequency regions: interface effects at low frequencies, mixed transport in the mid-range, and localized movements at high frequencies. Ce doping affects each region differently. It reduces available charge carriers by trapping them or changing their states. Ce doping causes complex changes in conductivity across all frequency regions. These changes are due to structural, defect, and grain boundary effects. Ce doping creates structural distortions because Ce ions are larger than Co^3+^. This causes lattice strain suppressing charge movements. The distorted structure creates tortuous paths hindering charge transport paths. Enhanced grain boundary scattering increases interfacial resistance, restraining the conductance plateau. The strain from Ce doping changes bond lengths and angles in the lattice. This affects charge carrier mobility. At low frequencies, strain makes grain boundaries harder to cross, reducing DC conductance. At high frequencies, short-range hopping (like Co^3+^ ↔ Co^2+^ transitions) dominates, but strain-induced disorder lowers their mobility and hence reduces high-frequency conductivity. Ce doping creates oxygen vacancies to balance charge; these vacancies help ionic conductivity by providing paths for oxygen ions to move. At low frequencies, oxygen vacancies gather at grain boundaries, changing their behavior and affecting the plateau. At low frequencies, conductivity is dominated by grain boundary resistance and long-range transport limitations. At moderate frequencies, the conductivity is through oxygen vacancies and short-range electronic hopping (Co^3+^/Co^2+^) whose relaxation times are affected by lattice strain and defects. The lattice strain causes the mobility and, hence, the conductivity to decrease at high frequencies.

**Fig. 8:**
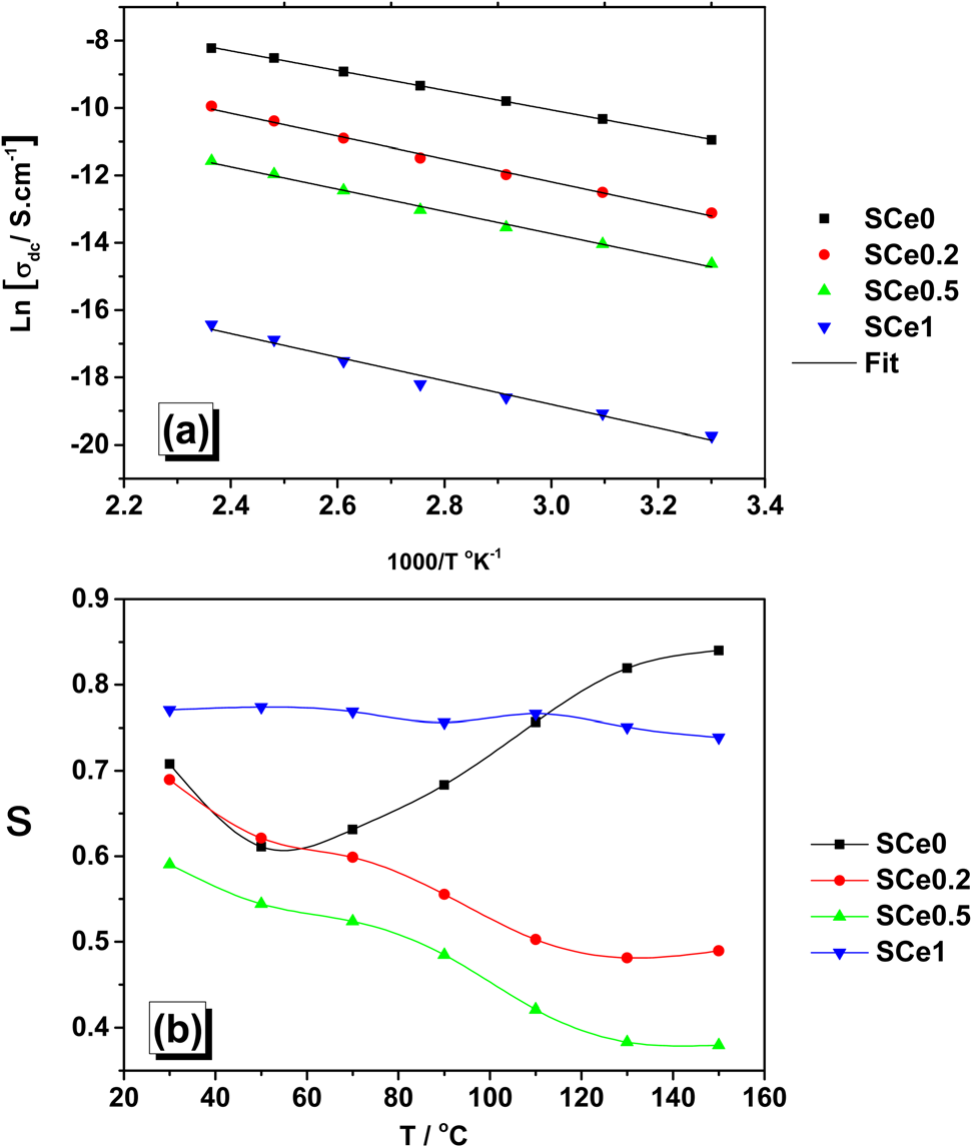
Arrhenius plots were used to extract activation energy (*E*_a_) from ln *σ vs.* 1000/*T* (a) and the temperature dependence of frequency exponent *s* (b).

Inspection of Fig. 8 and activation energies in Table 1 shows how the Ce inclusion affected the structure of the samples through increased activation energies with Ce doping. The increased activation energy at higher Ce doping could be perfectly attributed to lattice strain, oxygen vacancies, and structural disorder generated from Ce doping. The lattice strain distortions become stronger, creating more resistance to charge movement, and oxygen vacancies can trap charges instead of easing their motion and raising energy barriers, while structural disorder disrupts long-range transport paths, making it harder for ions or electrons to travel efficiently.

**Table 1:** The activation energy for all samples extracted from conductivity fitting

	SCe0	SCe0.2	SCe0.5	SCe1
Activation energy (*E*_a_) eV	0.258	0.286	0.291	0.302

The material shows different behaviors, and the conduction mechanism changes when Ce is added, as shown by Jonscher's power law exponent (*s*) variation with temperature. For SCe0, the *s* value goes down and then up as the temperature increases. This means charges move by hopping at low temperatures, and then change to a different way at high temperatures. Its low activation energy (0.258 eV) makes it easier for charges to move. When we add a little Ce (SCe0.2), *s* only decreases as temperature goes up. This tells us that charges move by hopping between barriers (correlated barrier hopping mechanism, CBH). The activation energy is higher (0.286 eV), so charges have a harder time moving. With more Ce (SCe0.5), *s* drops even more with temperature than that of SCe0.2. This means there's more disorder in the material, and charges get trapped more easily. The activation energy (0.291 eV) is higher too. At the highest Ce amount (SCe1), *s* barely changes with temperature. This means the way charges move stays almost the same, no matter the temperature. It has the highest activation energy (0.302 eV), showing the biggest changes in the material's structure. Adding more Ce makes the activation energy go up, which means charges have a harder time moving. Temperature affects *s* less when there's more Ce in the material. In SCe0, charges might start moving by tunneling (quantum mechanical tunneling mechanism, QMT) at high temperatures. In SCe1, charges might move by polaron transport or by hopping between defects. The more Ce we add, the more the material's structure changes, making it harder for charges to move around.

#### 3.5.3 The dielectric loss tangent

**Fig. 9:**
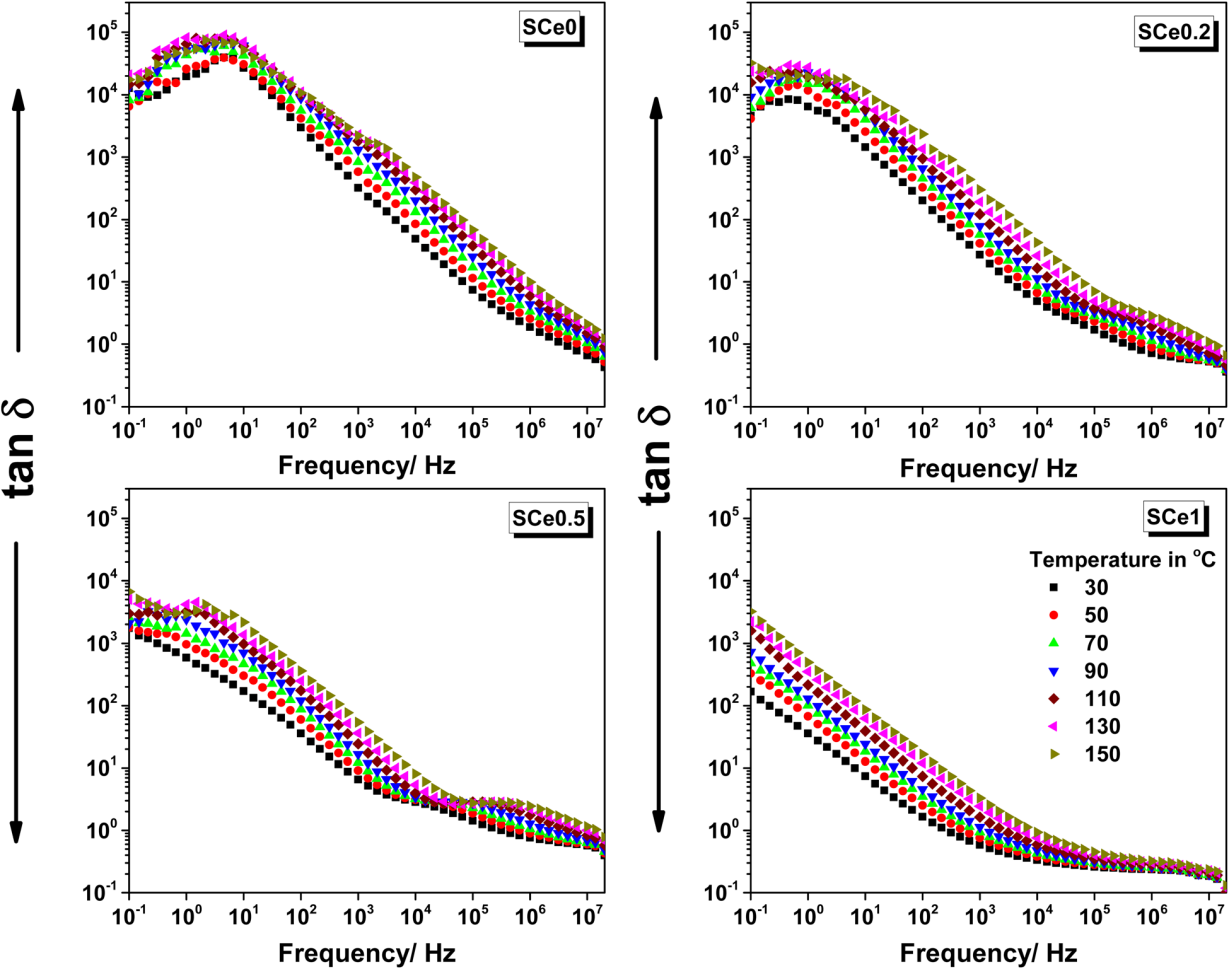
The dielectric loss tangent *vs.* frequency for all samples at different temperatures as indicated, from 30 °C to 150 °C, in steps of 20 °C.

The dielectric loss tangent is depicted in Fig. 9 for all samples *vs.* frequency and at different temperatures as indicated. The loss tangent curves show a peak at low frequency followed by a drop with frequency for samples SCe0, SCe0.2, and SCe0.5, while sample SCe1 starts the drop immediately from 0.1 Hz. The loss tangent for sample SCe0 shows the peak maximum at about 4.1 Hz with some symmetry around the maximum, followed by an almost vertical slope suggesting sudden onset. The curves maintain a linear relationship in the frequency range of 10^2^–10^5^ Hz across all temperatures, followed by a secondary peak or shoulder appearing at higher frequencies above 10^5^ Hz. Heating increases the loss tangent values at all frequencies and for all the samples. By heating the peak height increases non-linearly, and the slope becomes steeper with temperature. The linear relationship in the frequency range of 10^2^–10^5^ Hz shows parallel slopes in the temperature range of 30–70 °C, while in the higher temperature range of 90–150 °C, it shows progressively steeper slopes, indicating thermal activation.

Adding Ce causes the low-frequency peak's shape to show more broadness and height reduction with doping and the peak position to shift to lower frequencies progressively. This peak at 30 °C is centered at around 4.1, 0.45, and 0.15 Hz for samples SCe0, SCe0.2, and SCe0.5, respectively, while the peak center of sample SCe1 is shifted below 0.1 Hz and not seen in the measured scale. The secondary peak/shoulder at higher frequencies is very small and almost negligible in sample SCe0, while it becomes more prominent in both samples SCe0.2 and SCe0.5, and in sample SCe1 it disappears, suggesting that whatever mechanism caused it is suppressed or fully integrated into other relaxations. The secondary peak/shoulder is less noticed with heating for sample SCe0, while it becomes more evident in samples SCe0.2 and SCe0.5 on increasing the temperature. The relaxation behavior changes for the peak centered at low frequency *vs.* Ce content as follows: the apparent progressive increase in broadness suggests that sample SCe0 has a well-defined relaxation time, SCe0.2 has relaxation time distribution, SCe0.5 shows further broadening suggesting highly distributed relaxation, and more distributed relaxation for SCe1.

The loss tangent curve observations can be illustrated as follows. From the previous permittivity observations, we note three regions: a low frequency region that shows the first frequency dispersion due to interfacial polarization and charge transport, mid frequency region with almost frequency independent or plateau behavior. The high-frequency region is characterized by another frequency dispersion due to dipolar polarizations. Each dispersion shadows a peak in the loss tangent, which is noticed in its curves and in the same manner as in the permittivity. The first dispersion at low frequencies gets reduced with more Ce doping, and also, the second dispersion is barely noticed in the pure sample, with obvious presence in samples SCe0.2 and SCe0.5, and then disappears in sample SCe1. Here in the loss tangent curves, the low frequency peak comes from slow charge movement processes or conductivity contributions, and it shows interfacial polarization that originates from charges packed at grain boundaries. The peak height is related to the number of charges locked at the interfaces, while the peak position (frequency) shows the charges' response time or their speed. Broader peaks mean more different types of charge movements occurring together. Adding Ce causes more peak broadness because the presence of Ce introduces a more complex structure, different sites for charge entrapments and motions, more varied energy barriers, and more disrupted conduction pathways.

In terms of temperature effects, the increased heat promotes charges and dipole movements, which means more varied motions and, hence, more broad peaks. The non-linear increase with temperature is attributed to the increased number of both activated charge carriers and multiple processes activated together, along with the easier movement through barriers. The steeper slopes at high temperatures show that the charge movement is thermally activated, and the parallel slopes at lower temperatures (30–70 °C) are due to the same mechanism dominating, and the different slopes at higher temperatures (90–150 °C) are due to new mechanisms activated.

In terms of Ce content effects, adding Ce causes the low-frequency peak to shift to lower frequencies because larger Ce^4+^ ions make the structure more rigid, and because of the longer Ce–O bonds *vs.* Co–O bonds; these structure changes make more energy barriers and more difficult charge movement paths. The peak height gets smaller with more Ce due to fewer available charge carriers, as the structural changes disrupt conduction paths, and lead to more entrapped charges.

The secondary peak/shoulder shows up at higher frequencies related to dipole rotation or localized charge hopping. It is more visible in SCe0.2 and SCe0.5 because moderate Ce levels may create a balance between different mechanisms and offer the right amount of defects and charge carriers. The peak/shoulder disappears in SCe1 because the structure becomes too rigid, limiting dipole movement. The trapped charges can't participate due to the difficult conduction paths and the locking caused by the increased oxygen vacancies.

The samples SCe0.2 and SCe0.5 show a special balance in their properties. This Ce content introduces structural modifications that optimize dielectric properties by causing a modest permittivity and conductivity reduction coupled with significant improvements in the porosity and changing its structure nature into a flaky nature, providing a more specific surface area that would help greatly in other applications such as supercapacitor electrodes, gas sensors, and catalytic activities, and it will be our next project to assess the doping levels in such applications. The doping level of these samples creates enough defects and oxygen vacancies to be useful, but not too many. They have some structure changes but without complete disruption. There is also a good balance between trapped and moving charges. In SCe0.2, the structure starts to become flaky but retains some particle nature, with limited strain effects and a moderate number of defects. SCe0.5 shows a more developed flaky structure with more strain, but the strain is still manageable. It has more defects, but there are not too many. Both samples avoid the problems seen in other samples – SCe0 has too many moving charges, while SCe1 is too rigid with too many defects.

#### 3.5.4 The *Q*-factor analysis

In SCe0.2, the *Q*-factor starts high at low frequencies and drops sharply to a deep minimum of around 1 Hz. This low point in the *Q*-factor becomes broader and shifts to a higher frequency as the temperature increases from 30 °C to 130 °C. After the low point in the *Q*-factor, the *Q*-factor increases steeply and reaches a peak between 10^3^ and 10^4^ Hz. This peak shifts toward higher frequencies with temperature and reaches values close to 40. Above 10^5^ Hz, the *Q*-factor drops slowly and then flattens, showing no significant change with temperature. All the curves for different temperatures converge at high frequency. In SCe0.5, the *Q*-factor starts lower at low frequencies compared to SCe0.2 and shows a broader, more gradual low point in the *Q*-factor. The lowest point also shifts to higher frequencies as temperature increases. The increase in *Q*-factor is slower, and the peak occurs in a similar frequency range but with lower values, at around 25. The high-frequency tail flattens above 10^5^ Hz, where all temperature curves overlap. The peak shift and low point in *Q*-factor movement with temperature are also observed but less sharply than in SCe0.2. SCe0.5 curves show less spread between temperatures, and the slope of the *Q* increase is gentler than in SCe0.2. There is the same pattern for both samples: a dip in the *Q*-factor at low frequency, peaking, and leveling off at high frequency. SCe0.2 shows stronger frequency and temperature sensitivity than SCe0.5.

The sharp and deep low point in the *Q*-factor in SCe0.2 at low frequencies is due to strong interfacial polarization at well-defined grain boundaries. The XRD pattern is highly crystalline with no sign of the CeO_2_ phase, indicating a clean single-phase spinel structure. SEM reveals tight and equiaxed grains, which confirm uniform grain boundary response. FTIR bands are stable and sharp, reflecting low structural distortion. The steep increase in *Q* with frequency comes from a fast transition from interfacial polarization to dipolar relaxation. This matches the tan *δ* peaks and shoulder features that also shift with temperature. The high *Q* peak in SCe0.2 shows that dielectric loss is low while *ε*′ remains stable over a broad frequency range. The peak shifts to higher frequency with increasing temperature, indicating that the relaxation processes are thermally activated. The conductivity increases at the same frequency at which *Q* decreases, which is as expected since higher conduction at a higher frequency decreases *Q*.

**Fig. 10:**
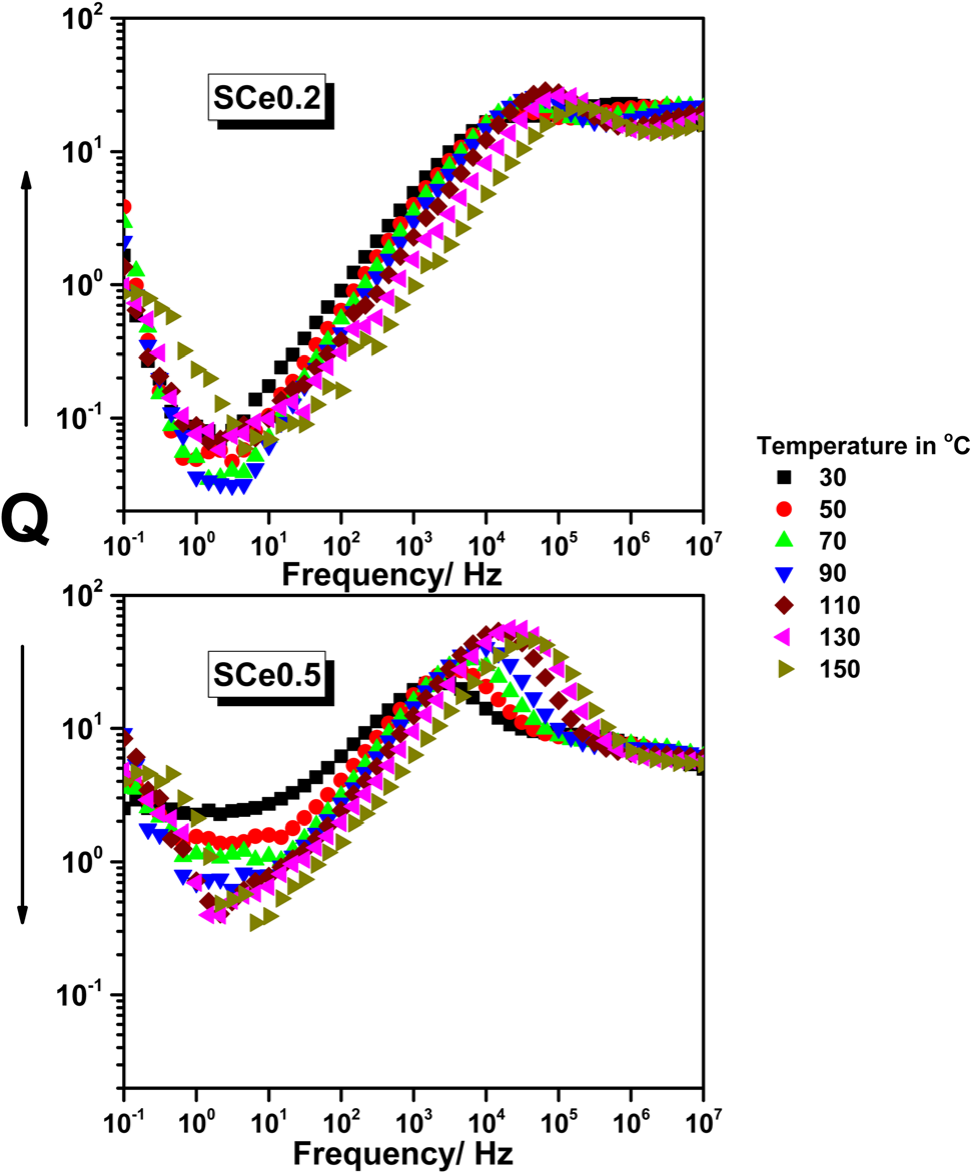
The *Q*-factor *vs.* frequency for samples SCe0.2 and SCe0.5 as representatives at different temperatures as indicated, from 30 °C to 150 °C, in steps of 20 °C.

In SCe0.5, the low-frequency low point in the *Q*-factor is broader, and the starting *Q* is lower. XRD shows a minor CeO_2_ phase and broader peaks, indicating some phase mixing. SEM micrographs depict grain morphology that is irregular and flaky, which decreases uniformity. The peaks for FTIR become distorted and shifted, validating greater lattice strain. These changes in structure make interfacial polarization become distributed, making the *Q* low point in the *Q*-factor broader and less sharp. The increase in *Q* with frequency is slower, and the maximum *Q* value is lower than in SCe0.2 due to increased dielectric loss. The peak shifts with temperature, but the curves are more compact and less responsive compared to those of SCe0.2. A conductivity hump in SCe0.5 occurs close to the *Q* maximum, indicative of a temperature-dependent balance between conduction and polarization. In the higher-frequency regime, all *Q* curves saturate as dipoles become unresponsive. High defect density and phase segregation in SCe0.5 make the effectiveness in polarization less, leading to lower *Q* and reduced frequency response. SCe0.2 is a better performing material from the aspect of dielectric behavior through being a uniform network with less defect density (Fig. 10).

The dielectric behavior of the samples is dependent on their structure and morphology.^54^ Grain shape,^55,56^ phase purity, and bond shifts can provide some explanations regarding the dielectric response. SEM, XRD, and FTIR show these changes clearly. The dielectric curves have three polarization mechanisms at different frequency regions, identified as interfacial, dipolar, and electronic. Each region is dependent on both frequency and temperature. Comparing samples links Ce content, structure, and dielectric performance. This helps in determining the best composition.

The sharp increase in the value of the dielectric permittivity (*ε*′) at low frequencies is linked with the presence of uniform and equiaxed grains as seen from SEM images, especially in SCe0 and SCe0.2. The flat *ε*′ region at moderate frequencies fits with the pure spinel phase confirmed by XRD and the absence of CeO_2_ peaks in these samples. The sudden change in the tan *δ* peak with increasing temperature also points towards a stable lattice structure, supported by well-distinguishable Zn–O and Co–O bands in FTIR. The sample SCe0.5 shows higher dielectric loss and broader tan *δ* peaks, which matches the noticed CeO_2_ peaks seen in XRD and the flaky, disordered grains seen in SEM. FTIR band shifts in SCe0.5 also show lattice distortion due to Ce doping. SCe0.2 shows a steeper increase in AC conductivity at high frequency, which means fewer traps and cleaner grain boundaries. In contrast, SCe0.5 shows a mid-frequency conductivity bump, which comes from trap release at irregular grain boundaries. The *Q*-factor peak is higher in SCe0.2, which reflects lower dielectric loss and a more stable *ε*′ due to its better structure. The dielectric behavior follows three main frequency regions. The low-frequency range (0.1–10 Hz), is governed by interfacial polarization as the dominant polarization mechanism. Here, a high value of *ε*′, a sharp tan *δ* peak, stable conductivity, and a low *Q*-factor characterize the region. On increasing temperatures, all these features shift towards higher frequencies. The dipolar relaxation seems to be the dominant mechanism in the mid-frequency range (10–10^4^ Hz). Here, *ε*′ stays almost constant (plateau), the tan *δ* curve forms a shoulder, conductivity starts to increase, and the *Q*-factor reaches its maximum. The peak position moves to a higher frequency with temperature and is sharper in SCe0.2 than in SCe0.5. In the high-frequency region (>10^4^ Hz), only electronic polarization remains active. In this region, *ε*′ drops, tan *δ* falls, conductivity follows a power law, and the *Q*-factor flattens. Curves for all samples and temperatures begin to converge in this region. Each composition shows different dielectric behaviors depending on the structure and Ce content. SCe0 has a pure spinel phase and uniform equiaxed grains. It shows the highest *ε*′ at low frequencies, the lowest dielectric loss (tan *δ*), a clear flat conductivity region up to 1 kHz, and the sharpest *Q*-factor features. SCe0.2 is also a single-phase but has slightly elongated grains. It has stable *ε*′ at moderate frequencies, well-defined tan *δ* peak shifts, a clear power-law region in conductivity, and a sharp, high *Q* peak. SCe0.5 shows some CeO_2_ phase in XRD and mixed grain shapes in SEM. *ε*′ becomes less stable, tan *δ* peaks are broader, conductivity is lower, and a bump appears in the mid-frequency range. Its *Q*-factor dip is broader, and the peak is lower. SCe1 shows strong CeO_2_ peaks and sponge-like flaky grains. It has the lowest *ε*′ and highest tan *δ*, with very low conductivity across the full range and minimal *Q* response. As Ce content increases, more trap states form, the lattice becomes more distorted, and dielectric performance drops. Loss increases, *ε*′ stability decreases, and *Q* becomes lower and flatter. Among all compositions, SCe0.2 gives the best overall balance—high and stable *ε*′, low tan *δ*, strong and clear conductivity behavior, and sharp *Q* features across the frequency range. It offers the most reliable dielectric response with good structural support.

### 3.6 Humidity sensor

**Fig. 11:**
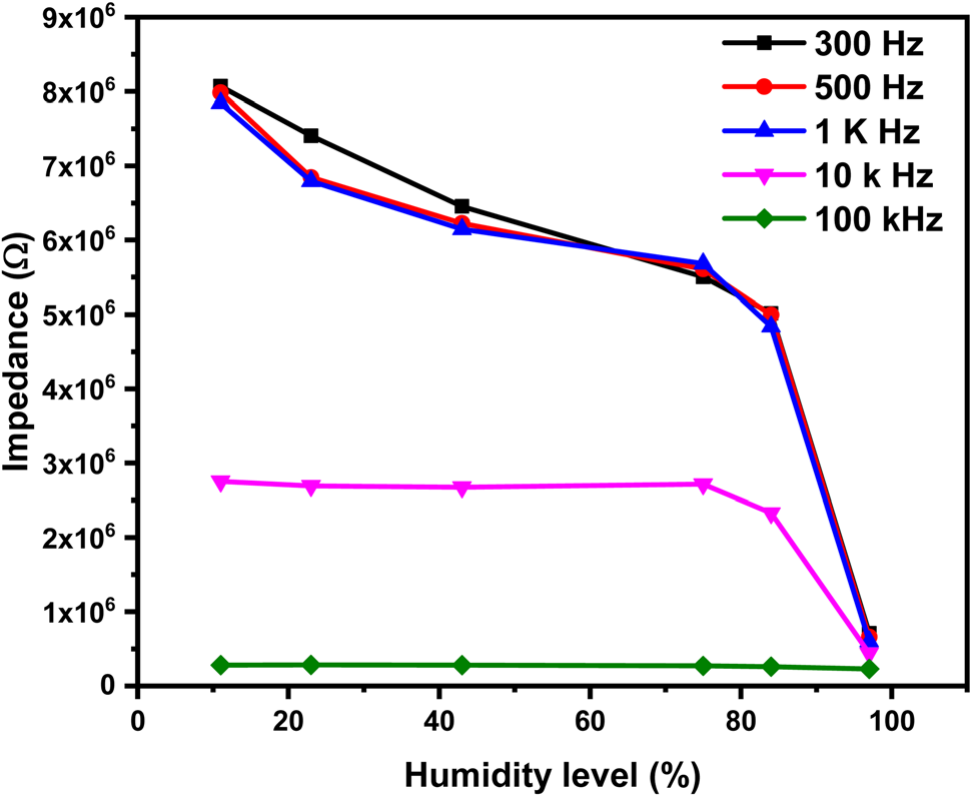
The impedance as a function of the humidity level of the 1% Ce sensor at different testing frequencies.

A humidity sensor is a device that suffers from a change in its physical properties when subjected to different levels of relative humidity. In this study, the focus is on monitoring the corresponding changes in impedance. The impedance value of the 1% Ce sensor is a frequency-dependent factor; hence, the sensor must be tested at different frequencies to determine the optimum testing frequency. The impedance variation as a function of relative humidity at different frequencies is shown in Fig. 11. The impedance *vs.* humidity curves in Fig. 11 showed a decrease in impedance as the amount of relative humidity increased. Moreover, the impedance variation is a function of testing frequency. It was recognized that the impedance variation decreases as the applied testing frequency increases, while it becomes insignificant at high testing frequency. This is related to the polarization of adsorbed water molecules. The polarizability of water molecules depends on the applied testing frequency. Water molecules are polar, and the polarizability of water molecules describes how the dipoles align with an externally applied electric field. At low frequency, the water molecules have enough time to align themselves with the applied field and thus affect the impedance. At high frequency, the applied electric field fluctuated rapidly, and the adsorbed water molecules do not have enough time to align with the applied electric field leading to a reduced polarization effect. The optimal testing frequency was determined to be 300 Hz, and all subsequent measurements were conducted at this frequency.

**Fig. 12:**
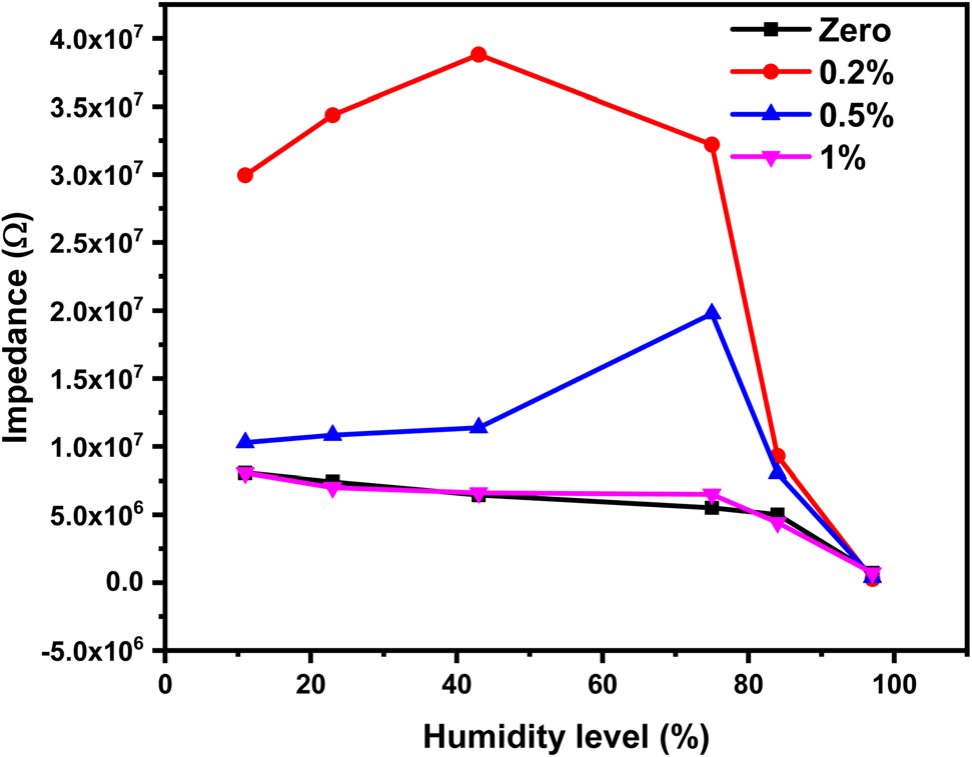
The response of all fabricated humidity sensors.

Fig. 12 shows the humidity-dependent impedance response of four fabricated sensors with varying cerium (Ce) doping concentrations (0%, 0.2%, 0.5%, and 1%). The undoped sensor (0% Ce) and the two lower-doped sensors (0.2% and 0.5% Ce) exhibit no clear trend in impedance variation up to 75% relative humidity (RH). However, above this threshold, their impedance decreases sharply, suggesting potential suitability for high-humidity applications. In contrast, the 1% Ce-doped sensor demonstrates a consistent decrease in impedance across the entire tested RH range (11–97%). This behavior may be attributed to enhanced porosity, as evidenced by the SEM images in Fig. 2, which reveal distinct pores in the 1% Ce sample. These pores likely facilitate water molecule adsorption, thereby improving humidity sensitivity. The response of the 1% Ce sensor can be divided into two regimes: (1) a linear region (11–84% RH), where impedance decreases gradually, and (2) a non-linear region (>84% RH), characterized by a rapid drop in impedance. This transition may reflect a shift in conduction mechanisms, such as the onset of capillary condensation or percolation-based ionic transport. Further investigation using complex impedance spectroscopy could elucidate the underlying processes, including the roles of grain boundaries and surface proton hopping, which will be discussed at the end of this section.

**Fig. 13:**
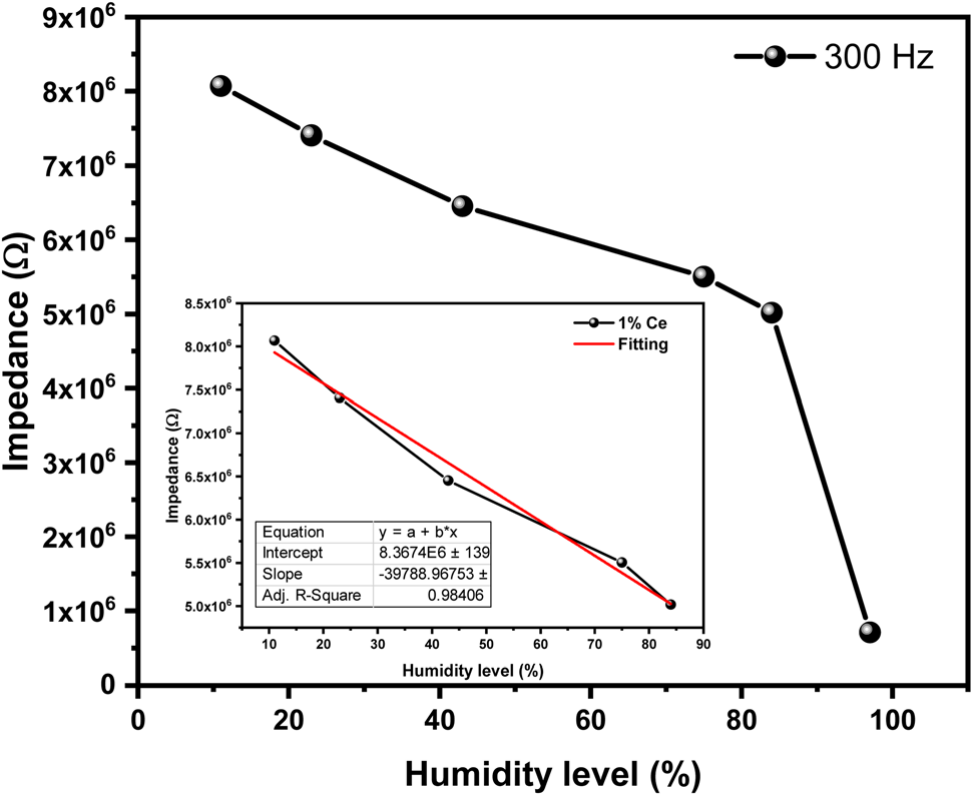
Impedance variation as a function of relative humidity (RH) for 1% Ce-doped sensors. The inset shows the linear fitting.

The two-regime response of the 1% Ce sensor shown in Fig. 13, which is linear at moderate relative humidity levels and non-linear at high humidity levels, reflects the general trends reported in porous ceramic-based humidity sensors. The 1% Ce-doped sensor reveals a linear relationship between impedance and relative humidity in the range of 11–84% RH (*R*^2^ = 0.98, see inset of Fig. 13). This linear behavior emphasizes the proportionality between the amount of adsorbed water molecules and charge carrier density. The linear relationship is highly encouraged and advantageous for humidity sensor applications. This sensor can be easily coupled with readout electronic devices without much-complicated calibration. The linearity of the 1% Ce sensor could arise from the uniform distribution of Ce–Ce-induced active sites that facilitate the adsorption of water molecules across the tested range (11–84%).

**Fig. 14:**
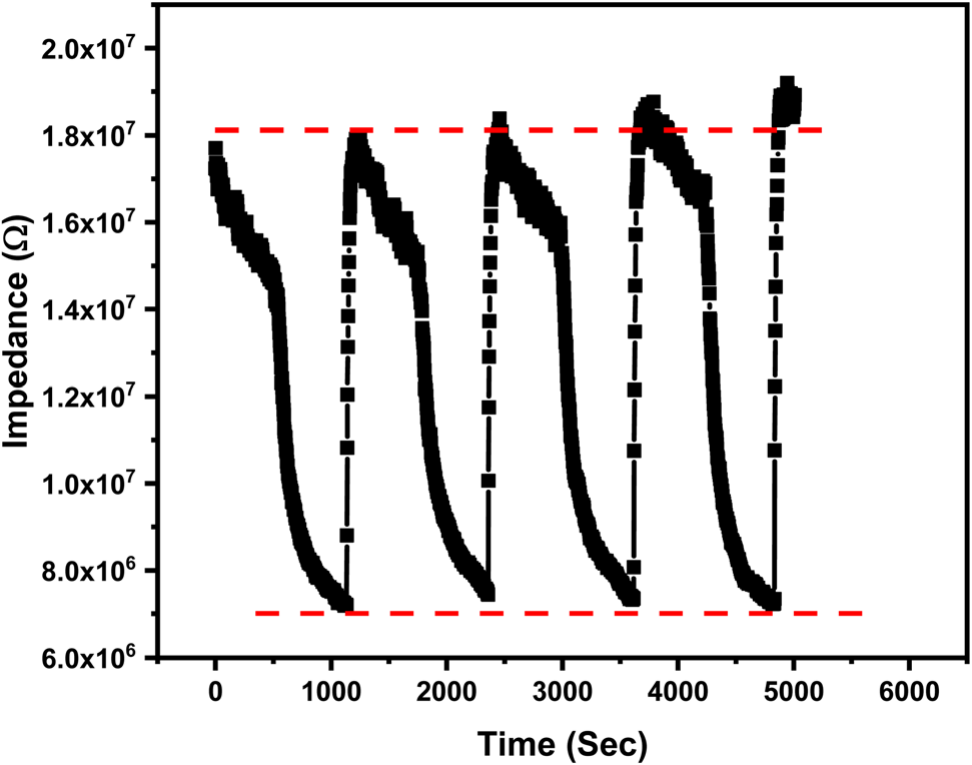
The repeatability of the 1% Ce sensor between two different humidity levels, 11% and 84%.

The sensitivity can be obtained from the slope in Fig. 13. The sensitivity of the 1% Ce doped sensor was found to be 0.4 MΩ/RH for humidity levels up to 84% and 0.33 MΩ/RH for humidity levels greater than 84%. The enhancement in sensitivity for humidity levels up to 84% could be due to the Ce-induced porosity, as confirmed by the SEM image in Fig. 2. The drop in sensitivity for humidity levels of more than 84% aligns with the Grotthuss mechanism, where bulk-like water layers minimize impedance variation. The repeatability of the 1% Ce sensor was evaluated by conducting humidity sensing measurements at two different humidity levels (11% and 84%) as shown in Fig. 14. The tested sensor demonstrates a consistent response with minimal variation. This repeatability confirms the reliability of the sensor to produce stable results.

Response time is defined as the time taken by the sensor to reach 90% of its maximum response upon exposure to a specific level of relative humidity, while recovery time is the duration required for the sensor to return to 90% of its baseline. The response and recovery times of the 1% Ce sensor are shown in Fig. 14. The response and recovery times were calculated and found to be 800 s and 20 s, respectively. It was observed that the tested sensor requires a prolonged time, while the water molecule release (uptake) takes place in a very short time. This could be due to the sensing material that traps water vapor gradually due to capillary condensation that requires much more time. More evaluation for humidity sensors has been performed by measuring the hysteresis. Hysteresis is an important parameter that deserves attention when dealing with humidity sensors. It measures the difference in a sensor impedance's value during the adsorption and desorption process.

Hysteresis (*H*) is evaluated by exposing the examined sensor (1% Ce) to a stepwise increase in relative humidity from 11% up to 97% flowed by exposing it to a stepwise decrease in relative humidity until it reaches 11% RH. Hysteresis is expressed as a percentage of the full-scale output. The hysteresis of the 1% Ce sensor is calculated using eqn (1) and shown in Fig. 15.1
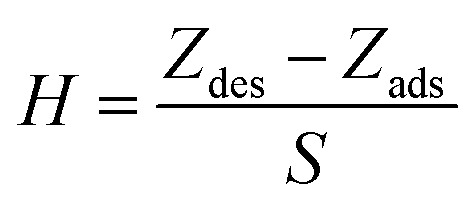
where *S* is the sensitivity, and *Z*_des_ and *Z*_des_ are the impedance values during the adsorption and desorption of water molecules, respectively.

**Fig. 15:**
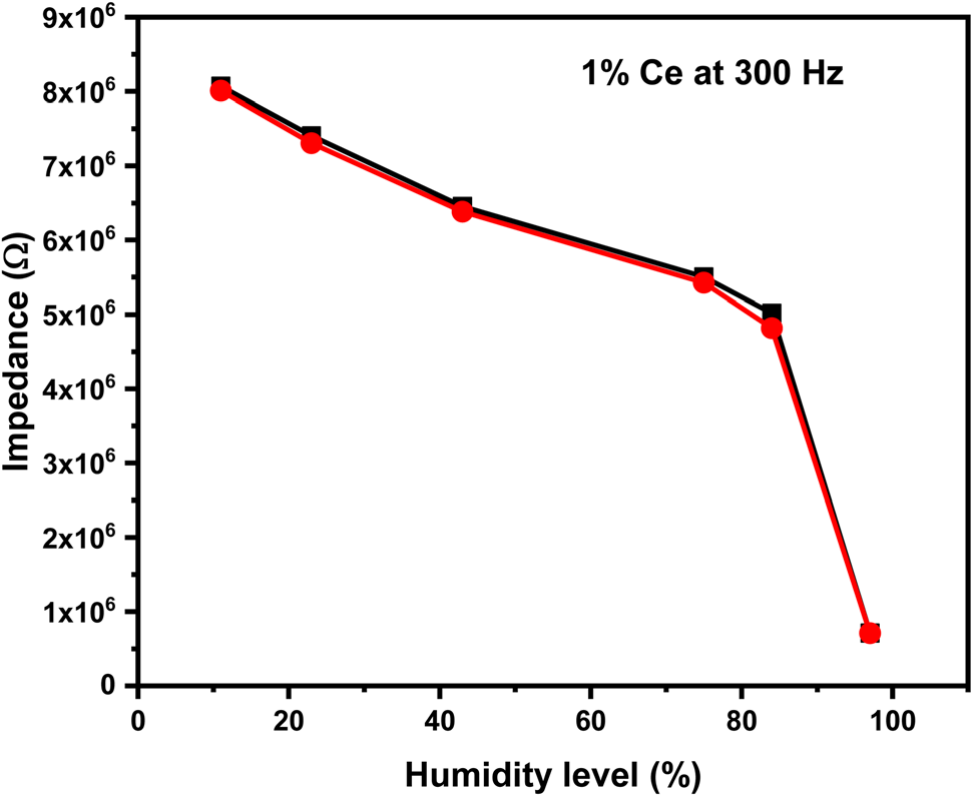
Hysteresis of the 1% Ce sensor measured at room temperature.

**Fig. 16:**
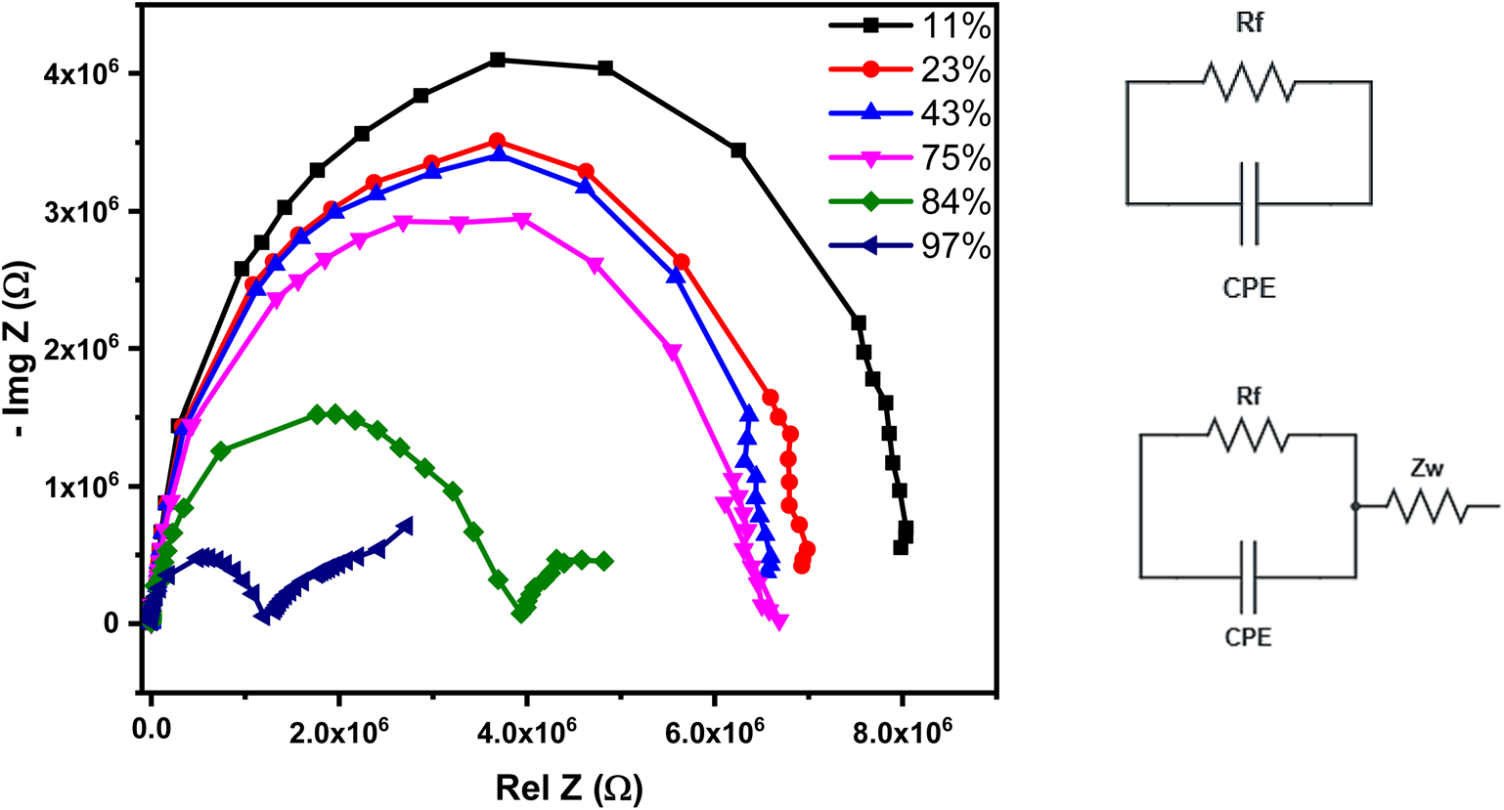
CIS of the 1% Ce sensor at different humidity levels and frequencies from 50 Hz up to 5 MHz, and the corresponding equivalent circuit.

The maximum hysteresis value of the tested sensor (1% Ce) was calculated to be 5%, which indicates a good reversibility that is essential for specific applications requiring high measuring precision. The humidity sensing mechanism was further evaluated using complex impedance spectroscopy (CIS) that correlates the sensor response with water molecule interaction at different testing frequencies. CIS with the corresponding equivalent circuit of the 1% Ce sensor at different humidity levels and frequencies from 50 Hz up to 5 MHz is illustrated in Fig. 16.

**Fig. 17:**
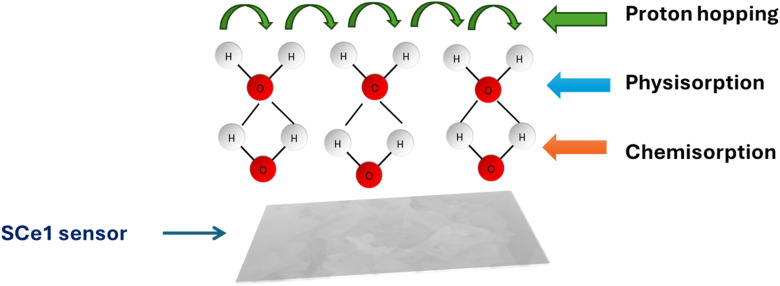
Schematic diagram of the humidity sensing mechanism.

The CIS curves of examined sensors reveal two different shapes: a semi-circle and semi-circle with a tail. These two shapes are related to two levels of relative humidity, *i.e.* the low to medium humidity level (11–75%) and high humidity level (84–97%). The water molecules can be visualized as layer-by-layer adsorbed water molecules. The first layer is bonded to the surface of the humidity sensor *via* a double hydrogen bond; hence, the charge carriers are restricted, and the charge carrier is transported through the sensor's intrinsic material (*e.g.*, grain boundaries in ceramics). As the humidity level increases, the curvature of the semi-circle decreases, indicating a reduction in charge carrier resistance. A further increase in the humidity level is accompanied by the adsorption of more water layers. This water layer is physically adsorbed. At this stage, the physically adsorbed water layer interacts with chemically bonded layers to produce a proton and hydronium. At a high humidity level, the Nyquist plot reveals a semi-circle coupled with a small tail at low frequency. The tail at low frequency arises from the Warburg impedance, where proton hopping becomes dominant. The transition from a semi-circle to a semi-circle with a tail is perfectly aligned with the Grotthuss mechanism, where the adsorbed water molecules form interconnected pathways for charge carriers. The humidity sensing mechanism is described schematically in Fig. 17. The humidity sensing properties of our fabricated sensor were compared to those of other fabricated sensors having approximately similar structures, as shown in Table 2.

**Table 2:** The comparison of relevant structures with the presented work for humidity sensing applications

Material	Response time (s)	Recovery time (s)	RH range %	Ref.
Sr–ZnFe_2_O_4_	19	81	10–95	57
Sn–NiFe_2_O_4_	44	180	10–95	58
ZnFe_2_O_4_	330	80	5–98	59
Mg_*x*_Zn_1−*x*_Fe_2_O_4_, *x* = 0.4	13	9	40–75	60
ZnCo_2_O_4_/PPy	8	7	0–97	61
SnO_2_–SiO_2_	14	16	11–96	62
Ni_*x*_Cu_0.8−*x*_Zn_0.2_Fe_2_O_4_, *x* = 0.4	320	230	5–98	33
ZnCo_(2−*x*)_Ce_*x*_O_4_, Ce = 1.0 wt%	800	20	11–97	This work

## 4 Conclusion

This paper presents the preparation of ZnCo_(2−*x*)_Ce_*x*_O_4_, (*x* = 0–2.0 wt%). The limited inclusion of cerium into the network reverts physicochemical assets explored by XRD, SEM, FTIR, VSM, and dielectric studies. XRD shows the major domination of zinc cobaltite with the cubic structure. The main diffraction peaks are allocated at 31°, 36°, and 65°, matching former reports. SEM results depict the transfer from the well-packed particle formation in the case of undoped cerium to the emergence and conjugation of particles with flaky shapes with increased distance in the doped cerium structures. FTIR displayed two core peaks residing at 557 cm^−1^ and 660 cm^−1^ attributed to Co–O and Zn–O stretching, respectively, while Ce insertion shifted these peaks to lower values, showing that the structure became less ordered. Charges accumulating at boundaries caused extremely high charge storage at low frequencies, leading to dielectric characteristics. Ce doping reduced charge storage and made it less temperature-sensitive. The conductivity was flat at low frequencies and subsequently increased according to the power law. Ce doping resulted in substantially reduced conductivity. Sample SCe0.5 demonstrated a distinctive conductivity bump because it had a nice balance of trapped and free charges. Finally, the optimized structure was investigated for humidity sensing at various humidity levels (11–84% RH). The repeatability, high sensitivity, and increased impedance variation features were reflected by the applied measurements. The response and the recovery times are 800 and 20 s, respectively. The validated physicochemical results and the humidity sensing performance nominate the composite for humidity sensing applications for industrial and technological sectors.

## Conflicts of interest

The authors declare that they have no known competing financial interests or personal relationships that could have appeared to influence the work reported in this paper.

## Data Availability

All data supporting this article is included in the manuscript.

## References

[cit1] Morsy M., Abdel-Salam A. I., Mostafa M., Elzwawy A. (2022). Micro Nano Eng..

[cit2] Farahani H., Wagiran R., Hamidon M. N. (2014). Humidity sensors principle, mechanism, and fabrication technologies: A comprehensive review. Sensors.

[cit3] Morsy M., Elzwawy A., Abdel-Salam A. I., Mokhtar M. M., El Basaty A. B. (2022). Diam. Relat. Mater..

[cit4] Alam N., Abid N., Islam S. S. (2024). ACS Appl. Nano Mater..

[cit5] Chellamuthu P., Savarimuthu K., Mohammed G. N. A., Arumugam T. (2025). IEEE Trans. Instrum. Meas..

[cit6] Wongsricha J., Sreejivungsa K., Thanamoon N., Harnchana V., Srepusharawoot P., Phromviyo N., Jarernboon W., Thongbai P. (2024). Sci. Rep..

[cit7] Zhang Y., Wu Y., Fu Y., xiang Jia Q., Zhang Z. (2024). Sensor. Actuator. B Chem..

[cit8] Malik P., Duhan S., Malik R. (2024). Mater. Adv..

[cit9] Omar F. S., Numan A., Duraisamy N., Bashir S., Ramesh K., Ramesh S. (2017). J. Alloys Compd..

[cit10] Tanwade S. B., Sapkal R. T., Deokate R. J. (2024). J. Mater. Sci. Mater. Electron..

[cit11] Heiba Z. K., Mohamed M. B., Abdellatief M., Arafat S. W., Sanad M. M. S., Badawi A. (2024). Phys. B.

[cit12] Ali R. N., Hassan M., Naz H., Qureshi W. A., Naveed A., Ali A., Liu Q. (2023). J. Clean. Prod..

[cit13] Gonçalves J. M., da Silva M. I., Silva M. N. T., Martins P. R., Nossol E., Toma H. E., Angnes L. (2022). Energy Adv.

[cit14] Rummaja I. D., Idris M. I., Napiah Z. A. F. M., Zamani Z. B., Ramlee R. H., Rashid M. (2024). J. Phys.:Conf. Ser..

[cit15] Muthukumaran K. P., Arjun V., Nithya A., Zhang J., Karuppuchamy S. (2025). Dalton Trans..

[cit16] Sitole S., Bilibana M. P., Ross N. (2025). J. Compos. Sci..

[cit17] Hameed M. M. (2023). AIP Conf. Proc..

[cit18] de la Luz Olvera M. , Morán-Lázaro J. P., López-Urías F., Muñoz-Sandoval E., Blanco-Alonso O., Guillén-Bonilla H., Guillén-Bonilla A., Rodríguez-Betancourtt V. M., Sanchez-Tizapa M. and Carreon-Alvarez A., Synthesis Characterization of Nanostructured ZnCo_2_O_4_ with High Sensitivity to CO Gas, in Nanostructured Materials-Fabrication to Applications, ed. M. S. Seehra, IntechOpen, Rijeka, 2017, p. 701

[cit19] Morán-Lázaro J. P., López-Urías F., Muñoz-Sandoval E., Blanco-Alonso O., Sanchez-Tizapa M., Carreon-Alvarez A., Guillén-Bonilla H., Olvera-Amador M. D., Guillén-Bonilla A., Rodríguez-Betancourtt V. M. (2016). Sensors.

[cit20] Amiri M., Mahmoudi-Moghaddam H. (2021). Microchem. J..

[cit21] Goudarzi M., Alshamsi H. A., Amiri M., Salavati-Niasari M. (2021). Arab. J. Chem..

[cit22] Abdali N. H. , Al-Rubaye S. H., Rabee B. H. and Abass K. H., in Journal of Physics: Conference Series, IOP Publishing, 2021, vol. 1818, pp. 12012

[cit23] Kamesh S., Athithya S., Harish S., Shimomura M., Navaneethan M., Archana J. (2024). Electrochim. Acta.

[cit24] Mohanty R., Parida K. (2024). Electrochim. Acta.

[cit25] Arulprakash G., Kareem A., Sellappan S. (2024). Int. J. Hydrogen Energy.

[cit26] Sun H., Miao Y., Wang G., Han X., Wang Y., Zhang Z., Luo C., Liu X., Xu C., Chen H. (2024). J. Energy Storage.

[cit27] Mahapatra P. L., Mondal P. P., Das S., Saha D. (2020). Microchem. J..

[cit28] Ateia E. E., Mohamed A. T., Morsy M. (2019). J. Mater. Sci. Mater. Electron..

[cit29] Gong L., Wang Z., Zhao J., Tang J., Li Z., Meng W., Qiu Z., Qin Y., Wang X., Zhang C., Zhang D. (2023). Chem. Eng. J..

[cit30] Kumar Y., Sharma A., Shirage P. M. (2017). RSC Adv..

[cit31] Pokhrel S., Jeyaraj B., Nagaraja K. S. (2003). Mater. Lett..

[cit32] Nakane T., Naka T., Nakayama M., Uchikoshi T. (2022). Sensors.

[cit33] Jeseentharani V., Reginamary L., Jeyaraj B., Dayalan A., Nagaraja K. S. (2012). J. Mater. Sci..

[cit34] Shimokawa K., Atsumi T., Harada M., Ward R. E., Nakayama M., Kumagai Y., Oba F., Okamoto N. L., Kanamura K., Ichitsubo T. (2019). J. Mater. Chem. A.

[cit35] Shaban Y., Alharbi N. A. (2022). Environ. Sci. Pollut. Res..

[cit36] Hasan Farooqi M. M., Srivastava R. K. (2017). J. Alloys Compd..

[cit37] Osaki T. (2018). J. Mater. Sci..

[cit38] Ouda E., Elzwawy A., Duraia E.-S. M. (2021). Appl. Phys. A.

[cit39] Foner S. (1956). Rev. Sci. Instrum..

[cit40] El Nahrawy A. M., Elzwawy A., Abou Hammad A. B., Mansour A. M. (2020). Solid State Sci..

[cit41] Aslam S., Shahzad Shifa M., Abbas Gilani Z., Noor ul Huda Khan Asghar H. M., Nauman Usmani M., Ur Rehman J., Azhar Khan M., Perveen A., Khalid M. (2019). Results Phys..

[cit42] El Nahrawy A. M., Mansour A. M., Elzwawy A., Abou Hammad A. B., Hemdan B. A. (2022). Environ. Nanotechnol., Monit. Manage..

[cit43] Elzwawy A., Talantsev A., Kim C. G. (2018). J. Magn. Magn. Mater..

[cit44] Ahmed Sheikh F., Abbas Gilani Z., Noor ul Huda Khan Asghar H. M., Khalid M., Mansoor Ali S., Khan N.-H., Ali Shar M., Kareem Khan A. (2023). J. Magn. Magn. Mater..

[cit45] Talantsev A., Elzwawy A., Kim C. (2018). J. Appl. Phys..

[cit46] Dippong T., Levei E. A., Cadar O., Deac I. G., Diamandescu L., Barbu-Tudoran L. (2019). J. Alloys Compd..

[cit47] Sheikh F. A., ul H. M. N., Khan Asghar H., Khalid M., Gilani Z. A., Ali S. M., Khan N.-H., Shar M. A., Mufti H., Alhazaa A. (2023). Phys. B.

[cit48] Mondal R. A., Murty B. S., Murthy V. R. K. (2014). Curr. Appl. Phys..

[cit49] Li G., Li X., Hao X., Li Q., Zhang M., Jia H. (2025). J. Environ. Sci..

[cit50] Chen L., Fleming P., Morris V., Holmes J. D., Morris M. A. (2010). J. Phys. Chem. C.

[cit51] Maciel A. P., Lisboa-Filho P. N., Leite E. R., Paiva-Santos C. O., Schreiner W. H., Maniette Y., Longo E. (2003). J. Eur. Ceram. Soc..

[cit52] V Skorodumova N., Ahuja R., Simak S. I., Abrikosov I. A., Johansson B., Lundqvist B. I. (2001). Phys. Rev. B.

[cit53] Manzoor A., Khan M. A., Khan M. Y., Akhtar M. N., Hussain A. (2018). Ceram. Int..

[cit54] Parveen A., ul H. M. N., Asghar H. K., Khalid M., Gilani Z. A., Aslam S., Saleem M., Shaikh F. A., Rehman J. (2019). Appl. Phys. A.

[cit55] Morsy M., Gomaa I., Abd Elhamid A. E. M., Shawkey H., Aly M. A. S., Elzwawy A. (2023). Sci. Rep..

[cit56] Elkatlawy S. M., Elzwawy A., Sakr A. A., Morsy M. (2024). J. Mater. Sci. Mater. Electron..

[cit57] Tiwari P. R., Singh R. P., Bharati K., Yadav A. C., Bhardwaj B., Yadav B. C., Singh A., Kumar S. (2024). J. Indian Chem. Soc..

[cit58] Jain M. K., Bhatnagar M. C., Sharma G. L. (1998). Appl. Phys. Lett..

[cit59] Jeseentharani V., George M., Jeyaraj B., Dayalan A., Nagaraja K. S. (2013). J. Exp. Nanosci..

[cit60] Patil S. N., Pawar A. M., Tilekar S. K., Ladgaonkar B. P. (2016). Sens. Actuators, A.

[cit61] Zhang D., Chen H., Zhou X., Wang D., Jin Y., Yu S. (2019). Sens. Actuators, A.

[cit62] Zhu Y., Chen J., Li H., Zhu Y., Xu J. (2014). Sens. Actuators, B.

